# Detection-Guided Keypoint Estimation for Humanoid Robots from Video Frames

**DOI:** 10.3390/s26185784

**Published:** 2026-09-11

**Authors:** Xuan Lou, Zhihuo Xu, Yuexia Wang

**Affiliations:** School of Transportation and Civil Engineering, Nantong University, Nantong 226019, China

**Keywords:** humanoid robot pose estimation, keypoint estimation, monocular video frames, detection-guided framework, heatmap regression, differentiable spatial to numerical transform, attention mechanism

## Abstract

**Highlights:**

**What are the main findings?**

- A detection-guided framework is developed for full-body 2D keypoint estimation of humanoid robots from monocular video frames.
- Robot-specific ROI normalisation, heatmap representation, DSNT decoding, and CBAM refinement improve localisation relative to direct coordinate regression.

**What are the implications of the main findings?**

- Task-specific local spatial modelling is more reliable than direct coordinate regression under limited robot annotations.
- The framework provides a lightweight basis for external monocular monitoring of humanoid robot poses.

**Abstract:**

Reliable estimation of humanoid robot body configuration from monocular RGB frames is useful for external monitoring in traffic, logistics, and service-robotics environments. However, direct transfer of human pose models is challenged by differences in body proportions, rigid surface appearance, joint morphology, and local texture, while robot-specific annotated data are often limited. This study presents a detection-guided framework for full-body 2D keypoint estimation from monocular video frames. A robot-specific detector first localises the target, and the detected box is expanded and normalised into a local region of interest (ROI) for keypoint recovery. Rather than treating the task as direct coordinate regression, the proposed integration uses ResNet18 feature extraction, convolutional block attention module (CBAM) refinement, heatmap-based keypoint representation, differentiable spatial to numerical transform (DSNT)-based continuous coordinate decoding, and skeleton-aware regularisation. A compact 13-keypoint annotation protocol is defined to describe the head, shoulders, elbows, hands, hips, knees, and feet. On the held-out test set, the proposed model reduces mean per-joint position error (MPJPE) from 27.98 px to 15.49 px relative to the ResNet18 direct-regression baseline and improves object keypoint similarity (OKS) from 0.80 to 0.96. Under the same 13-keypoint protocol, the proposed model also achieves a lower MPJPE than fine-tuned YOLOv8-Pose with detector-ROI input (18.85 px) and HRNet-W18 (30.82 px). Direct transfer of COCO-pretrained YOLOv8-Pose performs substantially worse, with an MPJPE of 201.50 px and an OKS of 0.28. These results support the effectiveness of robot-specific local spatial modelling and continuous coordinate recovery for monocular humanoid robot keypoint estimation under the evaluated limited-data setting.

## 1. Introduction

Visual perception is a fundamental capability for intelligent robotic systems. With the rapid development of deep learning, robotic vision has evolved from conventional geometric measurement and object localisation towards higher-level scene understanding, motion interpretation, and task-oriented decision making. Recent advances in large language models, three-dimensional vision, and artificial-intelligence-based control have further enhanced the ability of robots to perceive, reason, and interact with complex environments [1,2]. Meanwhile, vision-based robotic pose estimation, real-time six-degree-of-freedom pose tracking, monocular and binocular measurement, and lightweight visual grasping systems have demonstrated the increasing importance of visual perception in robot localisation, manipulation, navigation, and human–robot interaction [3,4,5,6]. These developments indicate that reliable visual interpretation of robot states is becoming an essential component of intelligent robotic systems.

Among different robotic platforms, humanoid robots present a particularly important and challenging case. Compared with wheeled or fixed-base robots, humanoid robots possess articulated body structures, limb-like motion patterns, and human-like interaction behaviours. Their pose states provide important cues for body orientation, locomotion intention, action recognition, and safety assessment. In practical applications such as traffic assistance, intelligent logistics, warehouse operation, service robotics, and public-space interaction, it is often necessary to estimate the motion state of a humanoid robot from ordinary camera observations. Compared with marker-based measurement, motion capture systems, or internal joint feedback, pose estimation from monocular video frames is easier to deploy, less dependent on platform-specific interfaces, and more compatible with existing surveillance or monitoring infrastructure [7,8]. Therefore, monocular video-frame-based pose estimation provides a practical and low-complexity solution for external perception of humanoid robots.

However, robust keypoint estimation for humanoid robots remains insufficiently explored. Most existing two-dimensional pose estimation methods have been developed for human bodies. Representative approaches such as DeepPose, OpenPose, OmniPose, and ViTPose have achieved substantial progress in human pose estimation, supported by large-scale annotated benchmarks such as Microsoft COCO (Common Objects in Context) [9,10,11,12,13]. Although humanoid robots share a coarse kinematic topology with humans, they differ substantially from human bodies in body proportion, joint configuration, surface material, local texture, and appearance consistency. These differences make direct transfer of human pose estimation models unreliable. In real monocular video frames, the problem is further complicated by scale variation, viewpoint changes, partial occlusion, motion blur, specular reflection, weak local texture, and cluttered backgrounds. As a result, keypoints on robot limbs and joints may be difficult to distinguish, especially when the robot occupies only a limited image region.

Existing studies related to robot visual perception have considered robot pose tracking, upper-body localisation, end-effector detection, and platform-specific keypoint estimation [14,15,16]. Nevertheless, full-body keypoint estimation for humanoid robots under monocular video-frame conditions has received comparatively limited attention. In particular, two issues remain important. First, directly applying human pose estimation models ignores the appearance gap between humans and humanoid robots. Second, training a pose model from scratch for robots is difficult because robot-specific datasets are usually much smaller than human pose datasets. These factors make it necessary to design a task-adapted framework that can make effective use of limited robot annotations while reducing interference from irrelevant image regions.

From a methodological perspective, humanoid robot keypoint estimation faces three major challenges. The first challenge is target localisation. If keypoints are regressed directly from the full image, the pose model must simultaneously handle background clutter, scale variation, and body-part localisation, which increases learning difficulty. The second challenge is spatial representation. Direct coordinate regression with a plain convolutional neural network may lose fine-grained spatial information, leading to unstable predictions under occlusion, weak texture, or reflective surfaces. The third challenge is structural consistency. Humanoid robot keypoints are not independent points but form an articulated skeleton. Without appropriate spatial and structural constraints, predicted keypoints may be locally inaccurate or geometrically implausible.

To address these problems, this paper proposes a detection-guided local keypoint estimation framework for humanoid robots from monocular video frames. The proposed method separates the task into two stages: target localisation and local keypoint recovery. A self-trained detector first localises the humanoid robot in the full image. The detected region is then cropped and resized into a normalised region of interest (ROI), within which a lightweight local pose network predicts 13 robot keypoints. This detection-guided design reduces background interference and allows the keypoint estimator to focus on robot body geometry in a more consistent local coordinate space.

Different from a plain ResNet18 direct-regression baseline, the proposed method does not simply regress keypoint coordinates from global image features. Instead, ResNet18 is used as a lightweight backbone for local feature extraction, and the network is enhanced by heatmap-based keypoint representation, differentiable spatial to numerical transform (DSNT)-based continuous coordinate decoding, and a convolutional block attention module (CBAM)-based feature refinement mechanism. The heatmap representation preserves local spatial response information around each keypoint. DSNT converts heatmap responses into continuous coordinates in a differentiable manner, avoiding the quantisation limitation of discrete heatmap peaks. CBAM further improves feature discrimination by adaptively emphasising informative channel and spatial responses. In addition, skeleton-aware regularisation is introduced to encourage geometrically reasonable keypoint configurations. Through these designs, the proposed framework combines the localisation advantage of object detection, the spatial modelling capability of heatmap regression, and the continuous coordinate accuracy of DSNT.

**Figure 1:**
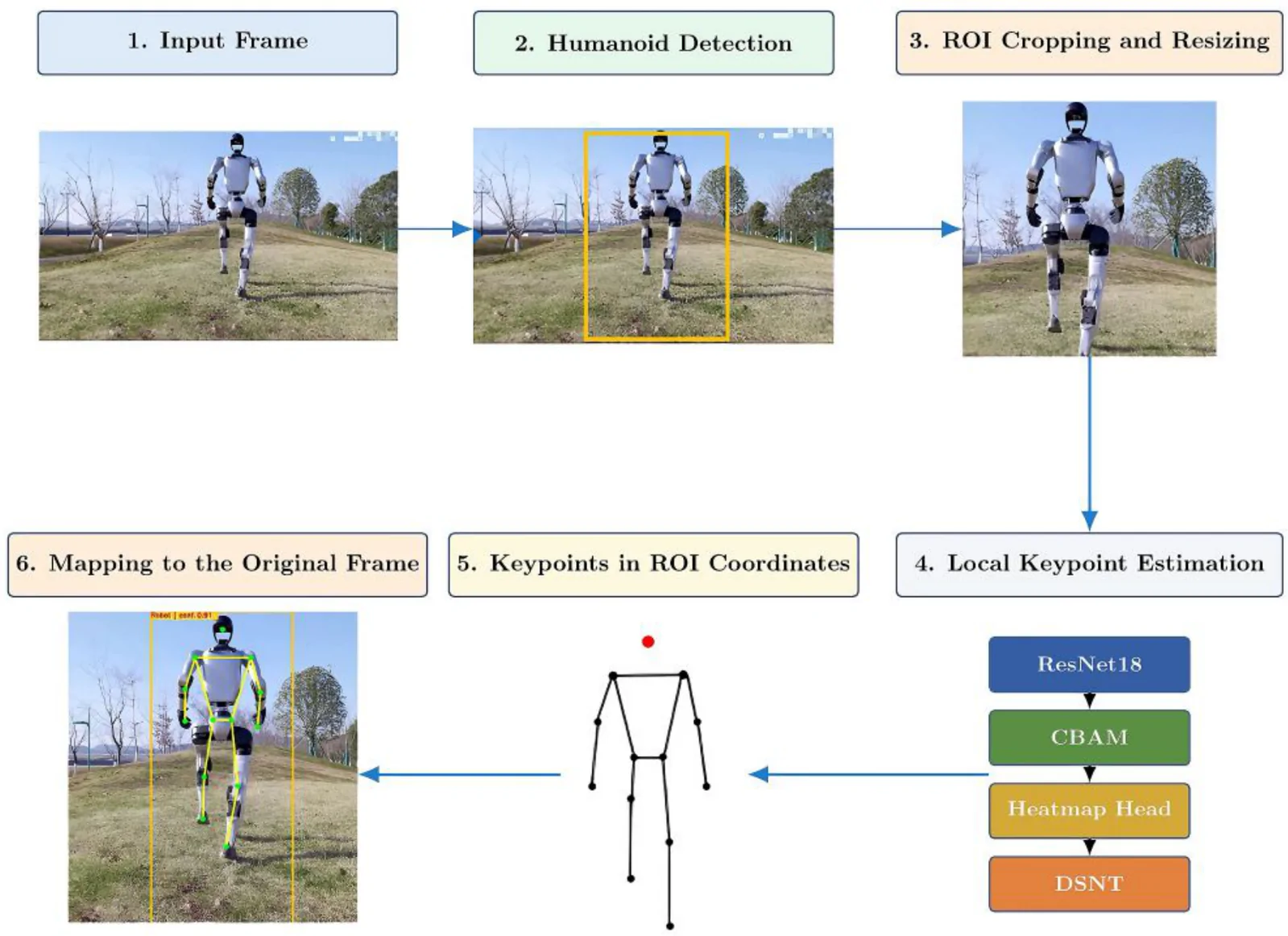
Overall pipeline of the proposed detection-guided local keypoint estimation method. The method first localises the humanoid robot in the input image, crops and resizes the detected region into a normalised region of interest, predicts 13 keypoints using the local pose estimation network, and maps the predicted keypoints back to the original image.

The overall pipeline of the proposed method is shown in Figure 1. Under the unified evaluation protocol, the proposed model achieves lower MPJPE than the ResNet18 direct-regression baseline, fine-tuned YOLOv8-Pose with detector-ROI input, and HRNet-W18, while detector-ROI YOLOv8-Pose attains a slightly higher PCK@0.20. These results support the use of robot-specific local spatial modelling for humanoid robot keypoint estimation from monocular video frames.

The main contributions of this paper are summarised as follows:

- A detection-guided local keypoint estimation framework is proposed for humanoid robot pose perception from monocular video frames. By separating robot localisation from local keypoint recovery, the framework reduces background interference and improves pose estimation under scale variation and cluttered scenes.
- A compact 13-keypoint annotation protocol is designed for full-body humanoid robot pose description. The selected keypoints cover the head, shoulders, elbows, hands, hips, knees, and feet, providing a task-relevant representation for body orientation, limb configuration, locomotion state, and coarse action interpretation.
- A robot-specific integration for monocular humanoid robot 2D keypoint estimation under limited annotations is developed by combining ResNet18 feature extraction, heatmap-based keypoint representation, DSNT-based continuous coordinate decoding, and CBAM-based attention refinement. This design improves spatial response modelling and coordinate accuracy compared with direct coordinate regression.
- Comprehensive experiments are conducted against directly transferred and fine-tuned YOLOv8-Pose models, HRNet-W18, and a ResNet18 direct-regression baseline under the unified 13-keypoint protocol. The results, together with extended ablation analysis, per-keypoint error evaluation, controlled ROI degradation, and backbone sensitivity analysis, verify the effectiveness of detection-guided local modelling, heatmap representation, continuous coordinate recovery, and attention-based feature refinement.

## 2. Related Work

### 2.1. Human Pose Estimation and Keypoint Representation

Human pose estimation provides an important technical foundation for humanoid robot keypoint estimation. Early deep learning methods such as DeepPose [9] formulated human joint localisation as a direct coordinate regression problem, demonstrating that convolutional neural networks can learn body joint positions from images. However, direct regression relies mainly on global feature representation and provides limited explicit modelling of local spatial responses around keypoints. To improve localisation accuracy, heatmap-based methods became widely adopted. Simple Baseline [17] showed that a convolutional backbone combined with deconvolutional heatmap heads can achieve strong performance through dense spatial supervision. The high-resolution network (HRNet) [18] further improved keypoint localisation by maintaining high-resolution representations throughout the network and repeatedly fusing multi-scale features.

Although heatmap regression improves spatial representation, conventional heatmap methods usually recover keypoint coordinates by selecting the maximum response location. This discrete operation may introduce quantisation errors, especially when the heatmap resolution is lower than the input image resolution. To address this issue, integral regression and differentiable spatial to numerical transform (DSNT) were proposed to convert heatmap distributions into continuous coordinates [19,20]. These methods preserve the advantages of heatmap-based spatial supervision while enabling differentiable coordinate recovery. Therefore, existing human pose estimation methods have established three representative technical routes: direct coordinate regression, heatmap-based keypoint localisation, and continuous coordinate decoding.

However, most of these methods are designed and trained for human bodies. Their assumptions are closely related to human body appearance, clothing texture, limb proportions, and large-scale human pose datasets such as COCO [13]. Humanoid robots share only a coarse skeletal topology with humans, while their surface materials, joint structures, body proportions, and local visual patterns can be substantially different. As a result, directly transferring human pose estimation models to humanoid robots may lead to unstable keypoint localisation. This motivates the development of robot-specific keypoint representation and local pose estimation strategies.

### 2.2. Vision-Based Robot Pose Estimation

Vision-based robot pose estimation has attracted increasing attention as robotic systems are deployed in more open and dynamic environments. Different from human pose estimation, robot pose estimation needs to handle rigid or semi-rigid mechanical structures, platform-specific body designs, and visual appearances that may vary significantly across robot types. Amini et al. [14] proposed a lightweight method for real-time 2D pose estimation of multiple humanoid robots. Their work focuses on simultaneous multi-robot perception and real-time operation. The present study considers detailed keypoint recovery for a detected humanoid target under limited annotations. Cho et al. [15] developed a full-body humanoid robot pose estimation method for a head-worn-camera telepresence setting, where visual pose estimation supports three-dimensional interaction. In comparison, this study uses external monocular video frames and directly evaluates full-body 2D keypoints in the image plane. Ban et al. [16] proposed HoRoPose for holistic robot pose estimation from RGB images with unknown internal states. Its objective extends to broader robot-state recovery, while the present study focuses on image-space keypoint localisation.

Recent studies have further explored more advanced robot pose modelling strategies. CtRNet [21] combined segmentation, keypoint detection, and differentiable geometric reasoning for camera-to-robot pose estimation. The detected keypoints support geometric pose recovery, while the final objective differs from the image-space 2D keypoint output considered in this study. RoboKeyGen [22] decomposes robot pose and joint-angle estimation into 2D keypoint detection and diffusion-based 3D keypoint generation. Its detected 2D keypoints serve as an intermediate representation for subsequent 3D pose and joint-angle recovery. In the present study, full-body 2D keypoints are the final output and are mapped back to the original image coordinate system. Heo et al. [23] constructed a diverse humanoid robot pose dataset and investigated full-body 2D pose estimation under sparse-data conditions. This setting is closely related to the present study. Their work places greater emphasis on robot diversity and sparse-data learning, while this study investigates detection-guided local estimation and continuous coordinate recovery under a customised 13-keypoint protocol. More recent studies further illustrate the diversity of humanoid robot pose-estimation settings. Wang et al. [24] combined HRNet-Epipolar heatmaps with a conditional random field robot model to recover absolute 3D humanoid poses from multi-view RGB images. This method relies on four camera views and targets 3D joint recovery. Young and Roberts [25] adapted YOLOv8 to the Unitree G1 robot under a platform-specific 21-keypoint protocol. Their setting is closer to monocular 2D keypoint estimation, although the model and annotation definition are specific to one robot platform. These differences in sensing conditions, output space, and keypoint definition prevent a direct numerical comparison with the present monocular 13-keypoint setting without substantial task conversion. Collectively, these studies demonstrate the feasibility of learning-based robot pose estimation while also illustrating that sensing configuration, output space, robot platform, and prior assumptions differ substantially across existing methods.

Nevertheless, existing robot pose estimation studies are often associated with specific robot platforms, camera configurations, task scenarios, or data construction strategies. Some methods focus on upper-body pose, end-effectors, camera-to-robot calibration, or three-dimensional pose recovery, while full-body two-dimensional keypoint estimation for humanoid robots from ordinary monocular video frames remains less explored. In particular, when only limited annotated robot data are available, directly training a general pose model or transferring a human pose model may be insufficient. This paper therefore focuses on a practical monocular video-frame setting and investigates a detection-guided local keypoint estimation framework for full-body humanoid robot pose perception.

### 2.3. Robot Pose Estimation with Additional Priors

Additional priors can improve the completeness and stability of robot pose estimation. Bultmann et al. [26] estimated mobile robot pose using multi-view RGB images from a network of external smart cameras, where keypoint detections were fused and reprojection errors were minimised. RoboPose [27] incorporated known robot models and rendering-based comparison into the pose recovery process, enabling the estimation of robot six-degree-of-freedom pose and joint angles from RGB images. These studies show that multi-view observations, geometric constraints, robot models, and internal state measurements can effectively reduce ambiguity in robot pose recovery.

However, the use of such priors usually requires additional hardware, accurate robot models, calibration procedures, or access to internal joint information. These requirements may limit deployment in ordinary video-monitoring scenarios, where only monocular images or video frames are available. In contrast, monocular full-body keypoint estimation imposes stricter constraints on model design because the method must infer robot pose from limited visual information without relying on multi-view geometry or internal robot states.

To address this more constrained setting, the proposed method introduces a detection-guided local estimation strategy. Instead of estimating keypoints directly from the whole image, the humanoid robot is first localised, and then keypoints are recovered within a normalised region of interest. This design reduces background interference and scale variation. In addition, heatmap representation, DSNT-based continuous coordinate decoding, and attention-based feature refinement are integrated to improve local spatial modelling and coordinate accuracy. Accordingly, the proposed framework targets a comparatively constrained setting in which full-body 2D keypoints are estimated from a single monocular RGB frame without requiring a predefined 3D robot model or internal joint-state input. Table 1 provides a concise comparison of representative vision-based robot pose-estimation methods. The comparison distinguishes sensing conditions, output spaces, robot types, dataset scale or evaluation scope, and the use of robot-specific priors. Numerical dataset size is reported where it is explicitly available and directly interpretable; otherwise, the table reports the dataset/evaluation scope because several studies combine synthetic and real data or use task-specific sequences that do not admit a meaningful one-to-one scale comparison.

The table highlights the heterogeneous objectives and assumptions of existing robot pose-estimation methods. The present study focuses on full-body 2D keypoint estimation from monocular RGB frames and does not require a predefined 3D robot model or internal joint-state input.

## 3. Proposed Method

### 3.1. Dataset Construction and Robot Keypoint Protocol

This study addresses two-dimensional pose estimation of humanoid robots from monocular video frames. The image samples were obtained by extracting representative frames from publicly available videos containing humanoid robots under different viewpoints, body postures, illumination conditions, camera distances, and background scenes. Since adjacent frames from the same video often have strong temporal similarity, the training, validation, and test subsets were separated at the video level. Continuous video segments were also avoided from being simultaneously assigned to different subsets. This strategy reduces the risk of data leakage and ensures that the test set provides a more reliable evaluation of generalisation ability.

A total of 640 humanoid robot images were manually annotated in this study using LabelMe (5.8.3) [28]. The dataset split is shown in Table 2. The training and validation subsets were divided at a ratio of 8:2, while an independent test set was reserved for final performance evaluation.

To describe the full-body configuration of humanoid robots, a customised 13-keypoint protocol was designed. The keypoint order is listed in Table 3. Let *K* denote the number of keypoints. In this study, K=13. For each annotated robot instance, the *k*th keypoint in the original image coordinate system is denoted as

(1) $$q_{k}=\left(x_{k},y_{k}\right),\;k=0,1,\ldots ,K-1,$$

where $x_{k}$ and $y_{k}$ are the horizontal and vertical pixel coordinates, respectively. A visibility mask $m_{k}$ is assigned to each keypoint, where $m_{k}=1$ indicates that the keypoint is visible and valid for training or evaluation, and $m_{k}=0$ indicates that it is invisible or not used.

The selected keypoints cover the main body joints and limb endpoints that are visually observable in monocular images. The head, shoulders, and hips describe the global orientation and trunk configuration of the robot, while the elbows, hands, knees, and feet characterise limb articulation, support state, and locomotion-related posture.

Compared with human bodies, humanoid robots usually exhibit more rigid components, weaker local texture, artificial joint structures, and larger appearance differences across platforms. Therefore, directly adopting a dense human pose annotation protocol may introduce unnecessary ambiguity and reduce annotation consistency. Fine-grained facial landmarks, fingers, and other small structures were not included because they are often absent, weakly visible, or inconsistent among different robot types. The proposed 13-keypoint protocol therefore provides a compact and task-oriented representation that balances pose descriptiveness, annotation reliability, and applicability to downstream robot state analysis.

**Figure 2:**
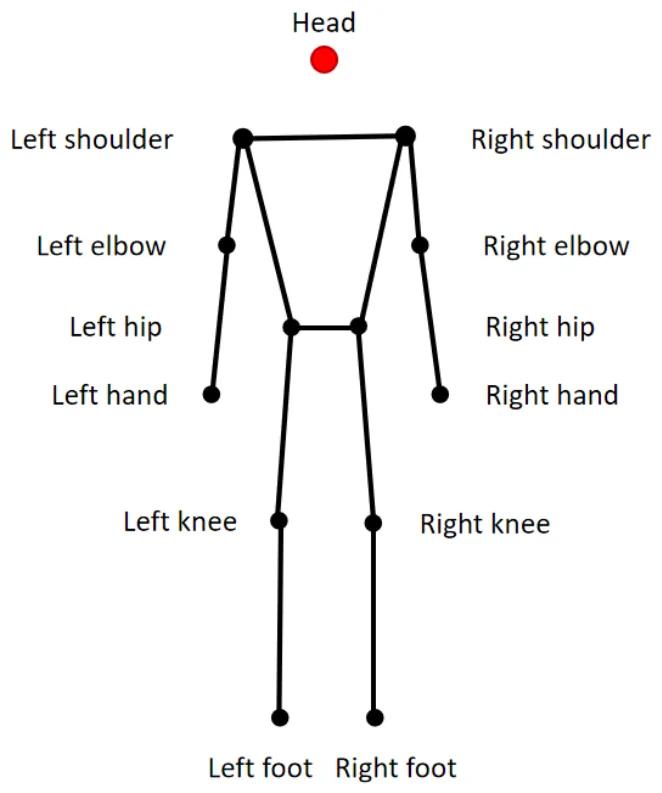
The 13 keypoint definitions and skeleton topology used in this study. Nodes represent manually annotated keypoints, and edges indicate the skeleton connections used for pose visualisation and structural regularisation.

The head point was manually annotated rather than inferred from facial landmarks, because facial regions are not always clearly defined on humanoid robots. All detection training, local pose estimation, evaluation, and ablation experiments were conducted under this unified keypoint definition. The skeleton topology is shown in Figure 2.

### 3.2. Background Diversification for Robust Pose Learning

Although the annotated frames extracted from public videos provide valid robot pose samples, their scene diversity is still limited. In practical applications, humanoid robots may appear in indoor rooms, outdoor roads, industrial sites, rescue scenes, and other complex working environments. If a model is trained only with a small number of original video backgrounds, it may learn background-dependent features rather than robot body structure, resulting in degraded performance under unseen scenes.

To improve robustness to environmental variation, background diversification was introduced during training. The aim is to preserve the annotated robot pose and keypoint geometry while varying the surrounding visual context. Specifically, the robot foreground is first normalised within its region of interest and then composited with different background images. Through this process, the pose label remains unchanged, while the model observes the same robot body structure under different scene conditions. This encourages the network to focus on robot shape, limb configuration, and local joint evidence rather than memorising specific background patterns.

**Figure 3:**
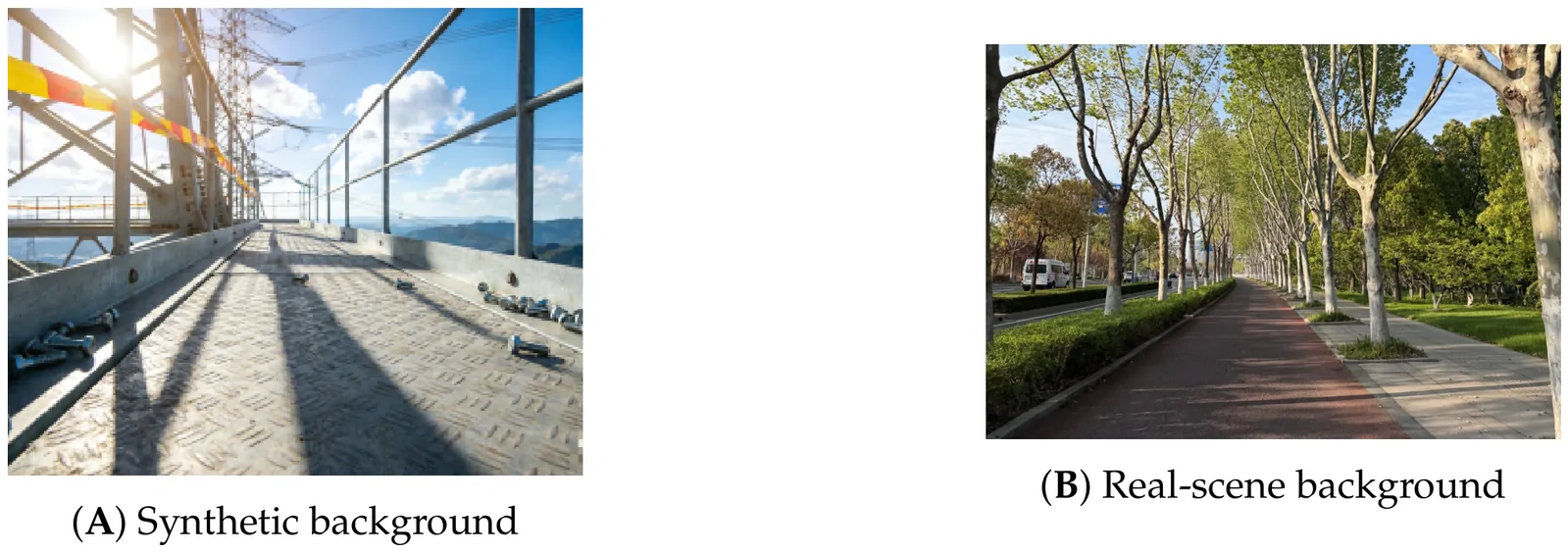
Examples of background images used for training augmentation. (**A**) A synthetic background; (**B**) a manually collected real-scene background. All background images were used only during training.

The background library consisted of manually collected indoor and outdoor images and 40 synthetic high-risk working scene backgrounds. The synthetic backgrounds were included to enrich the training distribution with visually challenging scenes that may be difficult to collect from real videos. Examples are shown in Figure 3. All background images were used only for training augmentation and were not included in the validation or test sets, ensuring that the evaluation was not biased by repeated background samples.

### 3.3. Overview of the Robot-Centred Pose Estimation Pipeline

The proposed method follows a robot-centred local pose estimation strategy. Instead of predicting all keypoints directly from the full image, the target robot is first localised, and then keypoint estimation is performed within a normalised region of interest. This design reduces the influence of image scale, camera distance, and background clutter, allowing the pose estimation network to concentrate on the relative spatial configuration of the robot body.

Given an input image, the front-end detector produces a bounding box enclosing the humanoid robot. The detected region is expanded by a small margin to preserve body extremities, cropped from the original image, and resized to a fixed input resolution. The local pose estimation network then predicts the 13 predefined keypoints in the normalised ROI coordinate system. Finally, the predicted keypoints are mapped back to the original image coordinate system for visualisation and quantitative evaluation.

This pipeline separates target localisation from local pose recovery. The detection stage provides a robot-centred observation window, while the pose estimation stage focuses on fine-scale keypoint localisation and structural consistency. Such a design is particularly suitable for humanoid robot images, where the target may occupy different image scales and appear in complex backgrounds. By operating in the ROI coordinate system, the model can learn a more stable mapping between robot body appearance and keypoint layout.

The local pose estimation network contains four functional components: feature encoding, attention-based feature refinement, heatmap representation learning, and differentiable coordinate decoding. The feature encoder extracts hierarchical visual cues from the normalised robot ROI. The attention module enhances keypoint-related responses and suppresses irrelevant background or texture interference. The heatmap head produces spatial response maps for the predefined keypoints, and the differentiable coordinate decoder converts these heatmaps into continuous keypoint coordinates. Therefore, the method combines the spatial interpretability of heatmap-based pose estimation with the numerical precision of coordinate regression.

The proposed framework is not designed around a specific backbone architecture. In this study, a compact convolutional encoder was adopted for efficient and reproducible feature extraction. The main methodological contributions lie in the robot-specific keypoint protocol, detection-guided ROI normalisation, perturbation-based training strategy, background diversification, heatmap to coordinate decoding, and skeleton-aware optimisation. These components jointly improve the robustness and structural plausibility of humanoid robot pose estimation under limited annotated data.

### 3.4. Detection-Guided ROI Normalisation and Perturbation Modelling

Accurate local pose estimation requires a reliable robot-centred image region as input. However, directly applying a general human detector, such as a COCO person detector, may lead to missed or inaccurate detections for humanoid robot targets because the object category, appearance distribution, body texture, and joint morphology differ from those of humans. To make the detection stage consistent with the target domain, this study trained a single class humanoid robot detector and used its output as the common ROI source for all pose estimation methods.

The detector outputs a bounding box enclosing the humanoid robot target. For detector evaluation, the reference bounding box was generated from the outer rectangle of the annotated keypoints and moderately expanded to cover the visible robot body. During inference, the detector processes the full image and returns candidate robot boxes. If multiple candidates are produced, the box with the highest confidence score is selected as the target box. The selected box is further expanded before being passed to the pose estimation network, reducing the risk of truncating hands, feet, or other limb endpoints near the ROI boundary.

To evaluate whether the detector can provide usable local regions for subsequent keypoint recovery, two detection-level metrics were used. Let *N* denote the number of effective labelled samples, $N_{\mathrm{hasdet}}$ denote the number of samples for which the detector produces at least one valid detection box, and $N_{\mathrm{matched}}\left(\tau \right)$ denote the number of samples whose selected detection box has an intersection over union not smaller than threshold τ with the reference bounding box. The detection rate is defined as

(2) $$\mathrm{DetRate}=\frac{N_{\mathrm{hasdet}}}{N}.$$

The ROI matching recall at IoU threshold τ is defined as

(3) $$\mathrm{Recall}@{\mathrm{IoU}}_{\tau }=\frac{N_{\mathrm{matched}}\left(\tau \right)}{N}.$$

In this study, τ was set to 0.20. This relatively relaxed threshold was adopted because the detector is used as a front-end ROI generator rather than as the final object detection output. Since the selected box is expanded before pose estimation, a detection is considered usable when it provides sufficient spatial overlap with the robot body for local keypoint regression.

The detector evaluation results are shown in Table 4. Among 140 effective labelled test samples, the detector produced valid boxes for all samples. No missed detection and no unmatched detection under ${\mathrm{IoU}}_{0.20}$ were observed. The detection rate and $\mathrm{Recall}@{\mathrm{IoU}}_{0.20}$ both reached 1.0000, showing that every test image produced a usable ROI at the deliberately relaxed overlap threshold used by the downstream pose pipeline. This result should not be interpreted as a high-precision object-detection benchmark, because the threshold was selected to assess ROI availability rather than box accuracy.

Based on this detector-level evaluation, the self-trained robot detector was used as the front-end localisation module in the proposed pipeline. All ROI-based pose-estimation methods used the same detector output so that differences in keypoint accuracy were not confounded by different front-end detectors.

Let the original image be denoted as *I*. Let the detected robot bounding box be

(4) $$B=(x_{1},y_{1},x_{2},y_{2}),$$

where $\left(x_{1},y_{1}\right)$ and $\left(x_{2},y_{2}\right)$ are the top left and bottom right coordinates of the detected box in the original image coordinate system, respectively. The width and height of *B* are defined as

(5) $$w_{B}=x_{2}-x_{1},\;h_{B}=y_{2}-y_{1},$$

where $w_{B}$ and $h_{B}$ denote the box width and height, respectively.

To preserve limb endpoints near the box boundary, the detected box is expanded before cropping. Let η denote the expansion ratio. The expanded box is defined as

(6) $$B^{e}=\left(x_{1}^{e},y_{1}^{e},x_{2}^{e},y_{2}^{e}\right),$$

where

(7) $$x_{1}^{e}=x_{1}-\eta w_{B},\;y_{1}^{e}=y_{1}-\eta h_{B},$$

(8) $$x_{2}^{e}=x_{2}+\eta w_{B},\;y_{2}^{e}=y_{2}+\eta h_{B}.$$

The expanded box is clipped to the image boundary before cropping. The local robot image cropped from *I* according to $B^{e}$ is denoted as $I_{B}$. The cropped region $I_{B}$ is then resized to the fixed network input size, producing the normalised ROI image $I_{r}$.

The ROI source differs between training and inference. During training, manually annotated keypoints are available, and a base ROI can therefore be constructed from the keypoint bounding rectangle. During inference, ground-truth keypoints are not available, and the ROI must be constructed from detector output. If the pose network is trained only with accurate ground-truth ROIs, it may adapt to overly ideal crops and become sensitive to detector shifts at inference. Directly using detector predictions during training may also introduce unstable box boundaries or occasional detection noise. Therefore, this study constructs a base ROI from the ground-truth keypoint box during training and applies controlled ROI perturbation to simulate inference-time detector variation.

Let $\left(c_{x},c_{y}\right)$ denote the centre of the base ROI, and let *w* and *h* denote its width and height, respectively. ROI perturbation is applied by changing the centre position, global scale, and aspect ratio:

(9) $${\tilde{c}}_{x}=c_{x}+\delta_{x}w,\;{\tilde{c}}_{y}=c_{y}+\delta_{y}h,$$

(10) $$\tilde{w}=w\left(1+\delta_{s}\right)\left(1+\delta_{a}\right),\;\tilde{h}=h\left(1+\delta_{s}\right)/\left(1+\delta_{a}\right),$$

where ${\tilde{c}}_{x}$ and ${\tilde{c}}_{y}$ are the perturbed ROI centre coordinates, $\tilde{w}$ and $\tilde{h}$ are the perturbed ROI width and height, $\delta_{x}$ and $\delta_{y}$ control horizontal and vertical centre shifts, $\delta_{s}$ controls global scale variation, and $\delta_{a}$ controls aspect ratio variation. The scale perturbation changes the ROI size, while the aspect ratio perturbation simulates detector box shape variation.

A training ROI is reconstructed from $\left({\tilde{c}}_{x},{\tilde{c}}_{y},\tilde{w},\tilde{h}\right)$. For the *k*th keypoint with original image coordinate $q_{k}=\left(x_{k},y_{k}\right)$, its normalised ROI coordinate is denoted as

(11) $$p_{k}=\left(p_{k}^{x},p_{k}^{y}\right),$$

where $p_{k}^{x}$ and $p_{k}^{y}$ are the horizontal and vertical coordinates in the normalised ROI coordinate system. They are computed as

(12) $$p_{k}^{x}=\frac{x_{k}-\left({\tilde{c}}_{x}-\tilde{w}/2\right)}{\tilde{w}},\;p_{k}^{y}=\frac{y_{k}-\left({\tilde{c}}_{y}-\tilde{h}/2\right)}{\tilde{h}}.$$

The main stage-wise ROI perturbation and augmentation settings are listed in Table 5. These settings are used in the training strategy described later.

### 3.5. Structure-Aware Local Pose Estimation Network

Given the cropped and resized ROI image $I_{r}$, the local pose estimation network predicts the 13 keypoints in the normalised coordinate space. Instead of directly regressing keypoint coordinates from global image features, the proposed network first learns keypoint-related heatmap representations and then converts them into continuous coordinates through differentiable decoding. This design combines the spatial interpretability of heatmap-based pose estimation with the numerical precision of coordinate regression.

**Figure 4:**
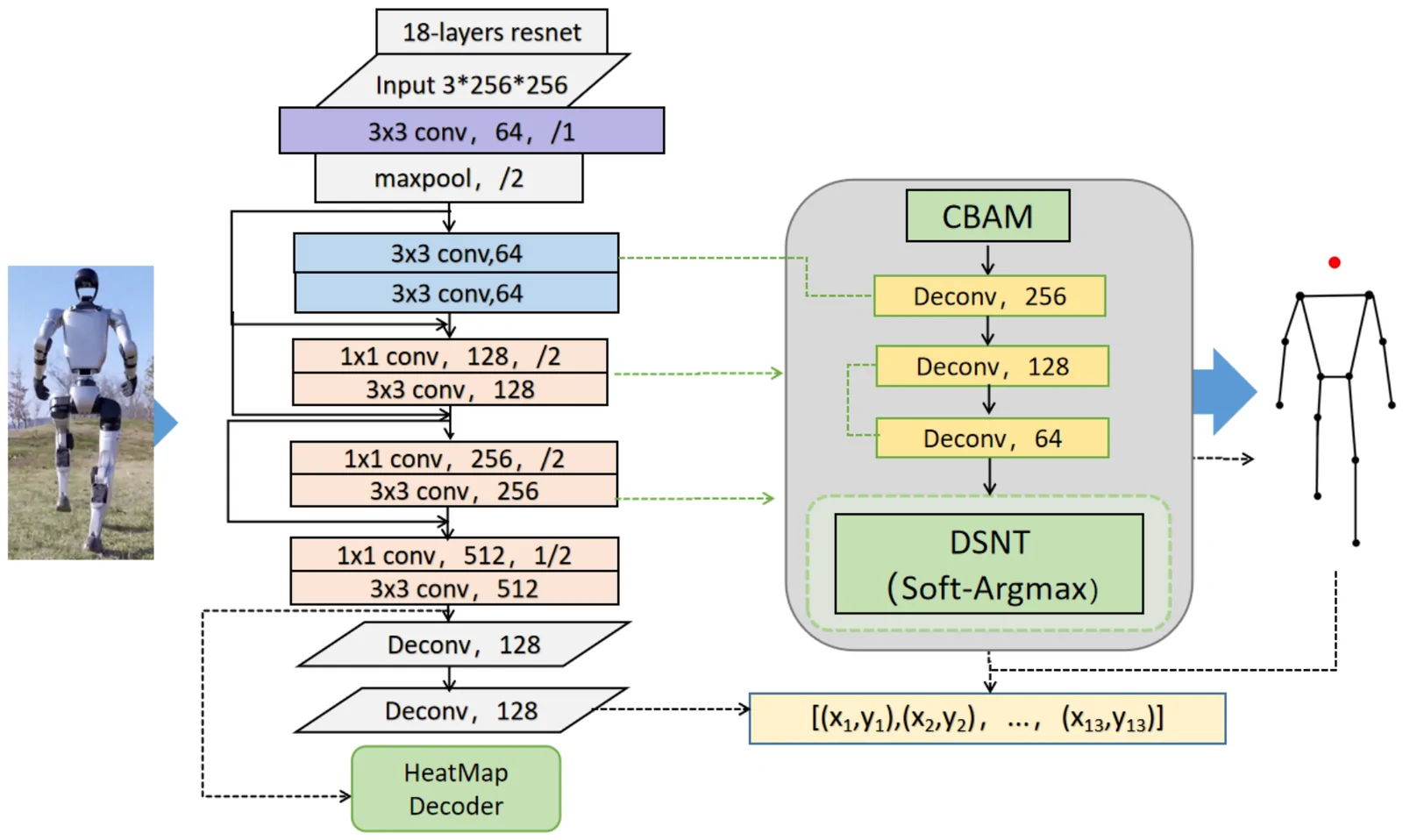
Architecture of the proposed local pose estimation network. The normalised ROI is processed by a lightweight feature encoder, followed by attention-based feature refinement, heatmap representation learning, and differentiable coordinate decoding. ResNet18 is used in the final configuration. Solid arrows indicate the main computational path; dashed lines are auxiliary visual guides and do not denote additional computational paths or skip connections. MobileNetV2 and ResNet34 replace only the feature backbone in the sensitivity experiment of Section 4.5.

The network consists of four main components: backbone feature encoding, attention-based feature refinement, heatmap representation learning, and differentiable coordinate decoding. The overall network structure is illustrated in Figure 4.

#### 3.5.1. Backbone Feature Encoding

The ROI image $I_{r}$ is first fed into a convolutional feature encoder to extract hierarchical visual representations of the robot body. Let S(·) denote the stem layer, and let $E_{i}\left(\cdot \right)$ denote the *i*th feature encoding stage, where i=1,2,3,4. The feature extraction process is written as

(13) $$F_{0}=S\left(I_{r}\right),F_{i}=E_{i}\left(F_{i-1}\right),\;i=1,2,3,4,$$

where $F_{0}$ is the output of the stem layer, $F_{i}$ is the feature map produced by the *i*th encoding stage, and $F_{4}$ is the final deep feature map.

In the implementation, ResNet18 [29] was adopted as a compact and reproducible feature encoder because of its moderate computational cost and stable hierarchical representation capability. However, the proposed framework is not specifically tied to ResNet18. The main methodological design lies in the robot-centred ROI normalisation, perturbation-based training strategy, heatmap to coordinate decoding, and skeleton-constrained optimisation.

#### 3.5.2. Attention-Based Feature Refinement

Humanoid robot keypoints are often located around small joint structures, limb endpoints, or weakly textured rigid parts. Background clutter and local appearance ambiguity may therefore interfere with keypoint localisation. To enhance keypoint-related responses, a convolutional block attention module (CBAM) [30] is inserted after the backbone output.

For the input feature map $F_{4}$, the channel attention map is defined as

(14) $$M_{c}\left(F_{4}\right)=\sigma \left(\mathrm{MLP}\left(\mathrm{AvgPool}\left(F_{4}\right)\right)+\mathrm{MLP}\left(\mathrm{MaxPool}\left(F_{4}\right)\right)\right),$$

where $M_{c}\left(F_{4}\right)$ denotes the channel attention map, σ(·) denotes the sigmoid activation function, MLP(·) denotes a multi-layer perceptron, and AvgPool(·) and MaxPool(·) denote spatial average pooling and spatial max pooling, respectively. The channel refined feature is

(15) $$F_{c}=M_{c}\left(F_{4}\right)⊙F_{4},$$

where $F_{c}$ is the channel refined feature map, and ⊙ denotes element-wise multiplication.

Spatial attention is then applied to $F_{c}$:

(16) $$M_{s}\left(F_{c}\right)=\sigma \left(f^{7\times 7}\left([{\mathrm{AvgPool}}_{c}\left(F_{c}\right);{\mathrm{MaxPool}}_{c}\left(F_{c}\right)]\right)\right),$$

where $M_{s}\left(F_{c}\right)$ denotes the spatial attention map, $f^{7\times 7}\left(\cdot \right)$ denotes a convolution with a kernel size of 7×7, ${\mathrm{AvgPool}}_{c}\left(\cdot \right)$ and ${\mathrm{MaxPool}}_{c}\left(\cdot \right)$ denote average pooling and max pooling along the channel dimension, and [·;·] denotes feature concatenation. The final attention refined feature is

(17) $$F_{a}=M_{s}\left(F_{c}\right)⊙F_{c},$$

where $F_{a}$ denotes the feature map after channel and spatial attention refinement.

#### 3.5.3. Heatmap Representation Learning

After attention-based feature refinement, a deconvolutional decoding head progressively restores spatial resolution and produces heatmap logits for all keypoints. Compared with direct coordinate regression, heatmap representation provides an intermediate spatial response map and allows the model to learn local keypoint evidence more explicitly.

Let $D_{l}\left(\cdot \right)$ denote the *l*th deconvolutional block, where l=1,2,3. The decoding process is written as

(18) $$H_{1}=D_{1}\left(F_{a}\right),$$

(19) $$H_{2}=D_{2}\left(H_{1}\right),$$

(20) $$H_{3}=D_{3}\left(H_{2}\right),$$

where $H_{l}$ denotes the feature map produced by the *l*th deconvolutional block. Each $D_{l}\left(\cdot \right)$ consists of transposed convolution, normalisation, and non-linear activation. A final 1×1 convolution produces the heatmap logits:

(21) $$Z={\mathrm{Conv}}_{1\times 1}\left(H_{3}\right)\in R^{K\times H_{h}\times W_{h}},$$

where *Z* is the heatmap logit tensor, K=13 is the number of keypoints, $H_{h}$ and $W_{h}$ denote the heatmap height and width, respectively, and ${\mathrm{Conv}}_{1\times 1}\left(\cdot \right)$ denotes a convolution with a kernel size of 1×1. The heatmap logit of the *k*th keypoint is denoted by $Z_{k}\in R^{H_{h}\times W_{h}}$.

#### 3.5.4. Differentiable Coordinate Decoding

To transform heatmaps into continuous coordinates, a differentiable spatial to numerical transform (DSNT) is adopted [20]. Conventional argmax decoding returns the discrete heatmap peak location and may introduce quantisation error. In contrast, DSNT computes the coordinate expectation over a normalised heatmap distribution, enabling end-to-end training and continuous coordinate recovery.

For the *k*th keypoint, the heatmap probability distribution is obtained by spatial softmax:

(22) $$P_{k}\left(u,v\right)=\frac{\exp(Z_{k}\left(u,v\right))}{\sum_{u^{\prime }=1}^{W_{h}}\sum_{v^{\prime }=1}^{H_{h}}\exp\left(Z_{k}\left(u^{\prime },v^{\prime }\right)\right)},$$

where $P_{k}\left(u,v\right)$ is the normalised probability at heatmap location (u,v), *u* is the horizontal heatmap index, *v* is the vertical heatmap index, and $Z_{k}\left(u,v\right)$ is the corresponding heatmap logit. The continuous normalised coordinates are computed as

(23) $${\hat{x}}_{k}=\sum_{u=1}^{W_{h}}\sum_{v=1}^{H_{h}}P_{k}\left(u,v\right)\;x\left(u\right),$$

(24) $${\hat{y}}_{k}=\sum_{u=1}^{W_{h}}\sum_{v=1}^{H_{h}}P_{k}\left(u,v\right)\;y\left(v\right),$$

where ${\hat{x}}_{k}$ and ${\hat{y}}_{k}$ are the predicted normalised horizontal and vertical coordinates of the *k*th keypoint, respectively. The functions x(u) and y(v) map the heatmap indices *u* and *v* to the normalised ROI coordinate system. The predicted coordinate of the *k*th keypoint is

(25) $${\hat{p}}_{k}=\left({\hat{x}}_{k},{\hat{y}}_{k}\right),$$

and the final network output is

(26) $$\hat{P}={\left\{{\hat{p}}_{k}\right\}}_{k=0}^{K-1},$$

where $\hat{P}$ denotes the set of all predicted keypoints in the normalised ROI coordinate system.

### 3.6. Joint Coordinate, Heatmap, and Structural Optimisation

The proposed model is trained using a joint objective that constrains coordinate accuracy, heatmap distribution, heatmap compactness, and skeleton geometry. This design avoids relying on a single coordinate regression loss and encourages the predicted keypoints to be both numerically accurate and structurally plausible.

The total loss is defined as

(27) $$L=\lambda_{coord}L_{coord}+\lambda_{hm}L_{hm}+\lambda_{shape}L_{shape}+\lambda_{bone}L_{bone},$$

where L denotes the overall training loss, $L_{coord}$ is the coordinate regression loss, $L_{hm}$ is the heatmap distribution loss, $L_{shape}$ is the heatmap shape constraint, and $L_{bone}$ is the bone-length regularisation loss. The coefficients $\lambda_{coord}$, $\lambda_{hm}$, $\lambda_{shape}$, and $\lambda_{bone}$ are non-negative weights used to balance these four loss terms.

The stage-wise loss weights used in the final model are summarised in Table 6. The coordinate term is kept as the main supervision signal with unit weight. The heatmap and shape terms are adjusted across stages to balance heatmap distribution learning and coordinate refinement. The bone-length term is kept constant to provide a mild but stable structural constraint.

For visible keypoints, the coordinate loss is applied to the continuous coordinates decoded by DSNT. Let $p_{k}=\left(p_{k}^{x},p_{k}^{y}\right)$ denote the ground-truth normalised coordinate of the *k*th keypoint, and let ${\hat{p}}_{k}=\left({\hat{x}}_{k},{\hat{y}}_{k}\right)$ denote the corresponding predicted coordinate. Let $m_{k}$ denote the visibility mask of the *k*th keypoint, and let $w_{k}$ denote the keypoint weight used to balance the contribution of different keypoints. The coordinate loss is defined as

(28) $$L_{coord}=\frac{\sum_{k=0}^{K-1}m_{k}w_{k}\;\ell_{\mathrm{SL}1}\left({\hat{p}}_{k}-p_{k}\right)}{\sum_{k=0}^{K-1}m_{k}w_{k}+\epsilon },$$

where ϵ is a small positive constant used for numerical stability, and $\ell_{\mathrm{SL}1}\left(\cdot \right)$ denotes the Smooth L1 loss. The Smooth L1 term is computed over the two coordinate dimensions:

(29) $$\ell_{\mathrm{SL}1}\left({\hat{p}}_{k}-p_{k}\right)=\rho \left({\hat{x}}_{k}-p_{k}^{x}\right)+\rho \left({\hat{y}}_{k}-p_{k}^{y}\right),$$

where ρ(·) is defined as

(30) $$\rho \left(z\right)=\left\{\begin{array}{cc} 0.5z^{2}, & |z|<1,\\ |z|-0.5, & \mathrm{otherwise}. \end{array}\right.$$

Here, *z* denotes the scalar coordinate error along one coordinate dimension.

In addition to coordinate supervision, a heatmap distribution loss is applied. Let $G_{k}\left(u,v\right)$ denote the Gaussian target heatmap distribution for the *k*th keypoint at heatmap location (u,v). The target distribution is centred at the ground-truth keypoint location in the heatmap coordinate system and normalised over all heatmap locations. The heatmap distribution loss is defined by the KL divergence:

(31) $$L_{hm}=\frac{1}{K}\sum_{k=0}^{K-1}m_{k}\sum_{u=1}^{W_{h}}\sum_{v=1}^{H_{h}}G_{k}\left(u,v\right)\log\frac{G_{k}\left(u,v\right)+\epsilon }{P_{k}\left(u,v\right)+\epsilon }.$$

This term encourages the predicted heatmap distribution $P_{k}\left(u,v\right)$ to match the target distribution $G_{k}\left(u,v\right)$ around each annotated keypoint.

Distribution matching alone does not necessarily guarantee a compact heatmap response. Therefore, an entropy-based heatmap shape constraint is further introduced to suppress overly dispersed responses. For the *k*th keypoint, the entropy of the predicted heatmap distribution is defined as

(32) $$H_{k}=-\sum_{u=1}^{W_{h}}\sum_{v=1}^{H_{h}}P_{k}\left(u,v\right)\log\left(P_{k}\left(u,v\right)+\epsilon \right),$$

where $H_{k}$ denotes the entropy of the predicted heatmap for the *k*th keypoint. The heatmap shape loss is then written as

(33) $$L_{shape}=\frac{\sum_{k=0}^{K-1}m_{k}H_{k}}{\sum_{k=0}^{K-1}m_{k}+\epsilon }.$$

A lower entropy value indicates a more concentrated heatmap response, which is beneficial for precise coordinate decoding.

To preserve skeleton geometry, a bone-length regularisation term is applied to the predefined skeleton edge set. Let E denote the set of skeleton edges shown in Figure 2. Each edge (i,j)∈E connects the *i*th and *j*th keypoints. The predicted and ground-truth bone-lengths for edge (i,j) are defined as

(34) $${\hat{b}}_{ij}={∥{\hat{p}}_{i}-{\hat{p}}_{j}∥}_{2},\;b_{ij}={∥p_{i}-p_{j}∥}_{2},$$

where ${\hat{b}}_{ij}$ and $b_{ij}$ denote the predicted and ground-truth bone-lengths in the normalised ROI coordinate system, respectively. Let $E_{v}\subseteq E$ denote the subset of skeleton edges whose two endpoints are both visible. Using the torso scale *r* as the normalisation factor, the bone-length loss is

(35) $$L_{bone}=\frac{1}{|E_{v}|}\sum_{\left(i,j\right)\in E_{v}}\left|\frac{{\hat{b}}_{ij}-b_{ij}}{r+\epsilon }\right|,$$

where $|E_{v}|$ denotes the number of valid visible skeleton edges. The torso scale *r* is computed as

(36) $$r={∥\frac{p_{1}+p_{2}}{2}-\frac{p_{7}+p_{8}}{2}∥}_{2},$$

where $p_{1}$ and $p_{2}$ correspond to the left and right shoulders, and $p_{7}$ and $p_{8}$ correspond to the left and right hips. This term constrains bone-length consistency without directly imposing directional constraints, thereby improving structural plausibility while preserving pose flexibility.

During inference, the predicted normalised keypoint coordinate ${\hat{p}}_{k}=\left({\hat{x}}_{k},{\hat{y}}_{k}\right)$ is mapped back to the original image coordinate system. Let the expanded inference ROI be $B^{e}=\left(x_{1}^{e},y_{1}^{e},x_{2}^{e},y_{2}^{e}\right)$, and let its width and height be

(37) $$w_{e}=x_{2}^{e}-x_{1}^{e},\;h_{e}=y_{2}^{e}-y_{1}^{e}.$$

The predicted pixel coordinate is then obtained as

(38) $${\hat{q}}_{k}=\left({\hat{q}}_{k}^{x},{\hat{q}}_{k}^{y}\right)=\left(x_{1}^{e}+{\hat{x}}_{k}w_{e},\;y_{1}^{e}+{\hat{y}}_{k}h_{e}\right),$$

where ${\hat{q}}_{k}$ denotes the predicted coordinate of the *k*th keypoint in the original image coordinate system.

### 3.7. Stage-Wise Training Strategy

The ROI image was resized to 256×256 pixels as the network input. The feature encoder was initialised with ImageNet pretrained weights to improve optimisation stability under limited annotated data. Online augmentation was applied in the ROI coordinate system, including horizontal flipping with corresponding left–right keypoint exchange, brightness and contrast perturbation, blur and noise, local occlusion, and background replacement.

The augmentation parameters followed the three-stage schedule shown in Table 5. This schedule was designed to gradually adjust the difficulty of ROI perturbation and local pose recovery. Stage 0 serves as a warm-up phase with moderate perturbations, allowing the network to learn stable keypoint responses. Stage 1 uses the strongest ROI perturbation and occlusion probability to expose the model to detector-like box shifts and challenging local observations. Stage 2 reduces the perturbation strength and removes background replacement, enabling the model to refine keypoint localisation under more stable ROI conditions.

This stage-wise strategy approximates the transition from robust representation learning under diverse detector box perturbations to accurate local coordinate recovery. It is particularly useful for small-scale humanoid robot pose datasets, where direct training with limited original backgrounds may lead to overfitting. The loss weights were also adjusted across stages, as shown in Table 6, so that the model first learns stable heatmap distributions and then gradually focuses more on accurate coordinate localisation.

### 3.8. Evaluation Metrics

Model performance was evaluated from four complementary perspectives: absolute localisation error, scale-normalised error, threshold-based keypoint accuracy, and overall keypoint similarity. All metrics were computed on visible keypoints.

Mean Per Joint Position Error (MPJPE) measures absolute keypoint localisation error in pixels. Let ${\hat{q}}_{k}=\left({\hat{q}}_{k}^{x},{\hat{q}}_{k}^{y}\right)$ denote the predicted pixel coordinate of the *k*th keypoint, and let $q_{k}=\left(x_{k},y_{k}\right)$ denote the corresponding ground-truth pixel coordinate. The Euclidean error of the *k*th keypoint is

(39) $$e_{k}={∥{\hat{q}}_{k}-q_{k}∥}_{2},$$

where $e_{k}$ denotes the pixel localisation error. Let $N_{v}$ denote the number of visible keypoints:

(40) $$N_{v}=\sum_{k=0}^{K-1}m_{k}.$$

MPJPE is then defined as

(41) $$\mathrm{MPJPE}=\frac{1}{N_{v}}\sum_{k=0}^{K-1}m_{k}e_{k}.$$

Since pixel-level error is affected by target scale, Normalised Mean Error (NME) is also used. Let *t* denote the torso scale in the original image coordinate system. It is computed as the distance between the shoulder midpoint and the hip midpoint:

(42) $$t={∥\frac{q_{1}+q_{2}}{2}-\frac{q_{7}+q_{8}}{2}∥}_{2}.$$

NME is defined as

(43) $$\mathrm{NME}=\frac{1}{N_{v}}\sum_{k=0}^{K-1}m_{k}\frac{e_{k}}{t+\epsilon }.$$

Percentage of Correct Keypoints (PCK) measures whether predictions fall within a given tolerance. Let α denote the tolerance threshold relative to the torso scale *t*. Under threshold α, PCK is defined as

(44) $$\mathrm{PCK}@\alpha =\frac{1}{N_{v}}\sum_{k=0}^{K-1}m_{k}I\left(e_{k}<\alpha t\right),$$

where I(·) is the indicator function, which equals 1 if the condition is true and 0 otherwise. This paper reports PCK@0.20.

Object Keypoint Similarity (OKS), originally used in COCO keypoint evaluation [13], is adopted to evaluate the overall matching quality of the keypoint set:

(45) $$\mathrm{OKS}=\frac{\sum_{k=0}^{K-1}m_{k}\exp\left(-\frac{e_{k}^{2}}{2A\sigma_{k}^{2}}\right)}{\sum_{k=0}^{K-1}m_{k}},$$

where *A* denotes the area of the ground-truth bounding box, and $\sigma_{k}$ denotes the tolerance parameter for the *k*th keypoint. Since this study uses a custom 13-keypoint protocol, the COCO 17-keypoint human parameters cannot be directly adopted. The base $\sigma_{k}$ values for the head, shoulder and hip, elbow and knee, and hand and foot keypoint groups are set to 0.08, 0.06, 0.07, and 0.09, respectively, and are uniformly multiplied by 1.4. The same $\sigma_{k}$ settings are used for all compared methods.

### 3.9. Training Parameters

The main training and inference settings used for Model D are summarised in Table 7. The stage-wise ROI perturbation and data augmentation settings have been reported in the preceding subsection.

These settings were fixed for the main experiments unless a different configuration is explicitly specified.

## 4. Experimental Results and Analysis

This section presents the experimental results under the unified 13-keypoint annotation protocol, detection-guided ROI construction process, and evaluation metrics described in the previous section. Section 4.1 reports the overall quantitative performance, pose-category results, qualitative outputs, and representative failure cases. Section 4.2 compares the proposed method with representative baseline methods, including a directly transferred human pose model and a robot-specific coordinate regression baseline. Section 4.3 analyses the contribution of the main components through step-wise ablation experiments. Section 4.4 evaluates the sensitivity of the proposed pipeline to ROI localisation errors. Section 4.5 examines the influence of backbone choice by retraining Model D with MobileNetV2 and ResNet34 under the same experimental protocol.

### 4.1. Overall Quantitative and Qualitative Performance

The overall test set performance of the direct coordinate regression baseline and the final model is reported in Table 8. Compared with the direct coordinate regression baseline, the final model reduces MPJPE from 27.98 pixels to 15.49 pixels, corresponding to a reduction of 44.6%. NME decreases from 0.11 to 0.06. PCK@0.20 increases from 0.85 to 0.97, and OKS increases from 0.80 to 0.96. Taken together, the four metrics show a consistent improvement over direct coordinate regression in both absolute and scale-normalised localisation accuracy. Because these values are aggregate test-set results, they are interpreted together with the pose-category, qualitative, and per-keypoint analyses below rather than as evidence of unrestricted cross-scene generalisation.

To examine performance across different robot poses, the test samples were divided into walking and turning categories according to their body configurations. The current test set was extracted from motion-oriented video sequences. Static poses were absent from this set and will be included in future dataset expansion.The category-specific results are reported in Table 9.

Walking poses yield lower localisation errors and higher keypoint accuracy than turning poses. MPJPE increases from 13.89 px to 22.06 px for turning poses, while PCK@0.20 decreases from 0.978 to 0.931 and OKS decreases from 0.970 to 0.941. This degradation is consistent with the greater limb overlap and reduced endpoint visibility that occur during body rotation.

**Figure 5:**
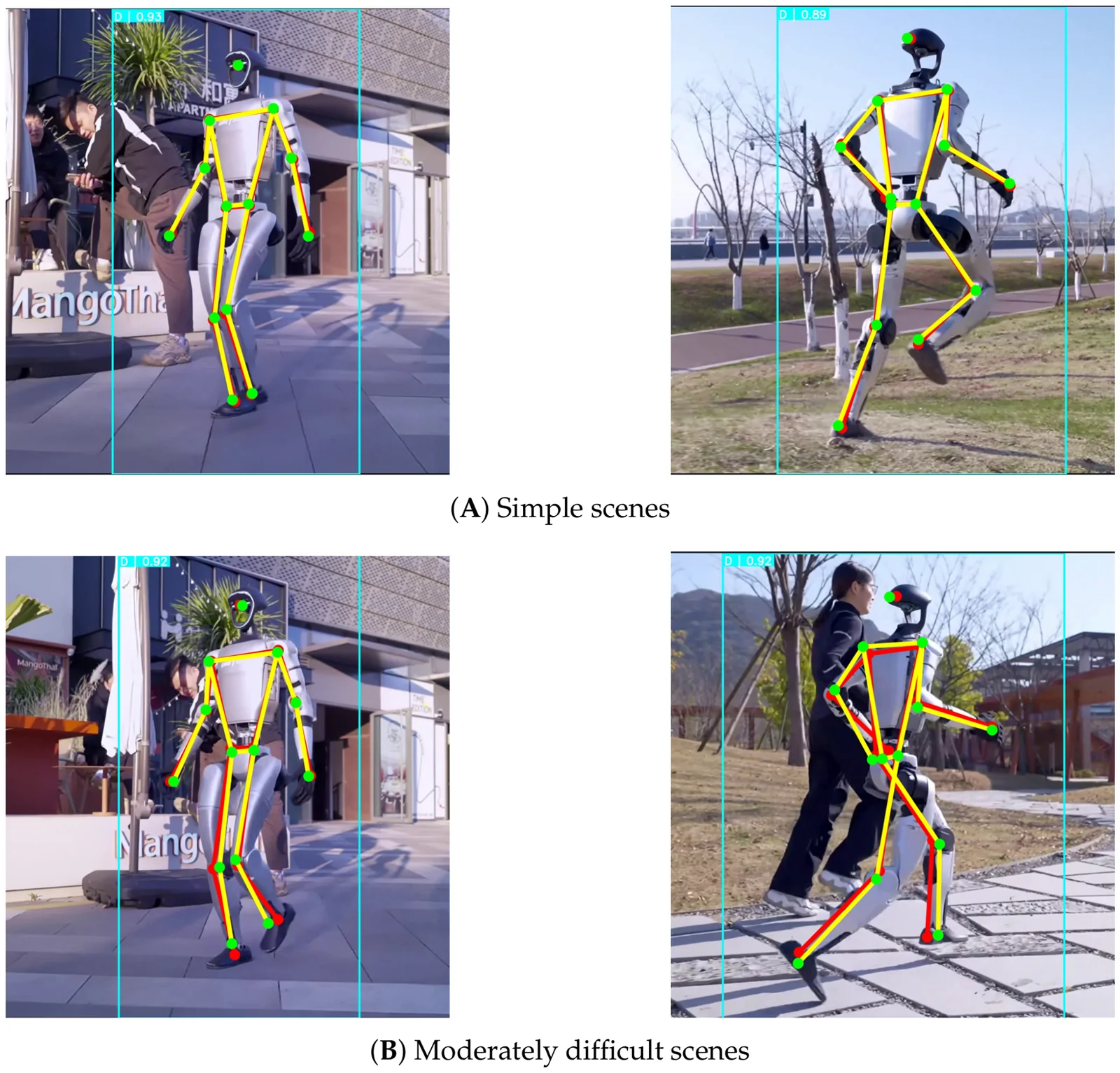

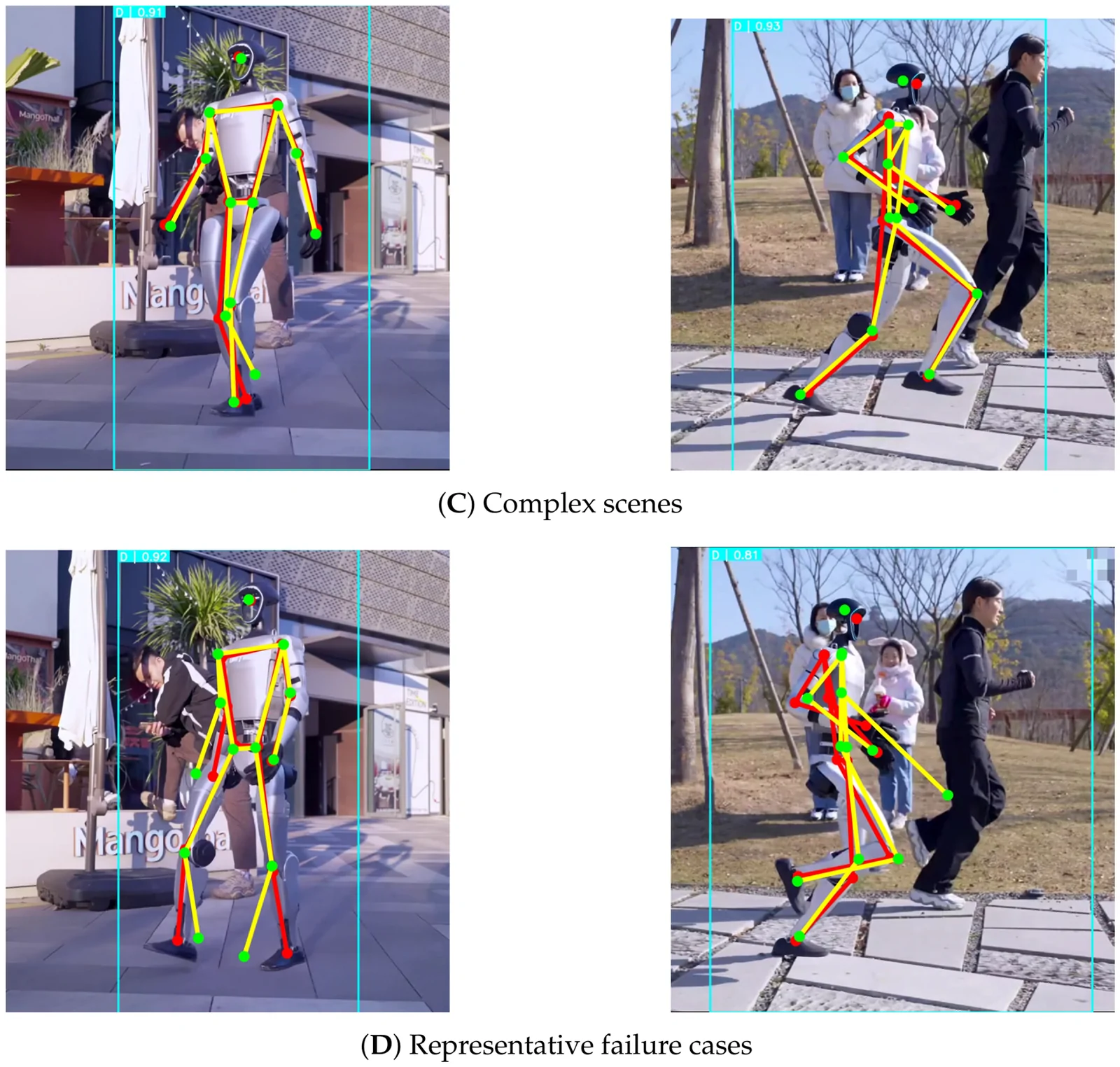
Qualitative pose estimation results under different scene complexities and representative failure cases. (**A**) Simple scenes with clear robot visibility and limited background interference; (**B**) moderately difficult scenes with scale variation, limb motion, partial interference, or less favourable viewpoints; (**C**) complex scenes with stronger occlusion, nearby distractors, endpoint ambiguity, or challenging viewpoints; (**D**) representative failure cases with inaccurate endpoint localisation. Red keypoints and skeletons denote ground truth, while green keypoints and yellow skeletons denote model predictions. Qualitative pose estimation results under different scene complexities and representative failure cases. The first failure case shows inward displacement of both foot keypoints. The second shows a hand prediction displaced forward relative to the corresponding elbow.

Figure 5 presents representative predictions under different scene conditions. Groups A–C show successful results with increasing visual difficulty, and Group D presents two representative failure cases. For visual analysis, the test samples were grouped according to scene complexity, including simple scenes, moderately difficult scenes, and complex scenes. The scene complexity was mainly determined by background clutter, target scale variation, viewpoint change, partial occlusion, nearby distractors, and the visibility of limb endpoints. These scene labels were used only for qualitative analysis and were not used as separate training or testing categories.

In simple scenes, the robot body is clearly visible, the background interference is limited, and the model can recover complete skeletons with accurate limb connectivity. In moderately difficult scenes, moderate scale variation, limb motion, partial interference, or less favourable viewpoints may occur. Under these conditions, the head, shoulders, and hips remain stable, while some limb endpoints may show small local deviations. In complex scenes, stronger occlusion, nearby distractors, endpoint ambiguity, or challenging viewpoints make keypoint localisation more difficult. Although hand and foot keypoints may show larger offsets in these cases, the overall skeleton topology remains largely coherent. These observations indicate that the main errors are not caused by global pose collapse but are concentrated in flexible endpoint regions under complex scene conditions.

Group D presents two representative failure cases. In the first case, both foot keypoints shift toward the body centreline even though the detector provides an ROI aligned with the robot and the upper-body skeleton is recovered correctly. The error is concentrated near the feet, where weak local texture, ground reflections, and similar left–right lower-leg appearance may reduce endpoint discriminability.

In the second failure case, the hand keypoint shifts forward relative to the corresponding elbow, producing an implausible forearm length. Background clutter remains inside the detected ROI, and the arm has limited contrast with nearby regions. These visual conditions may introduce ambiguous local evidence even when detector-guided cropping has removed most of the global background.

**Figure 6:**
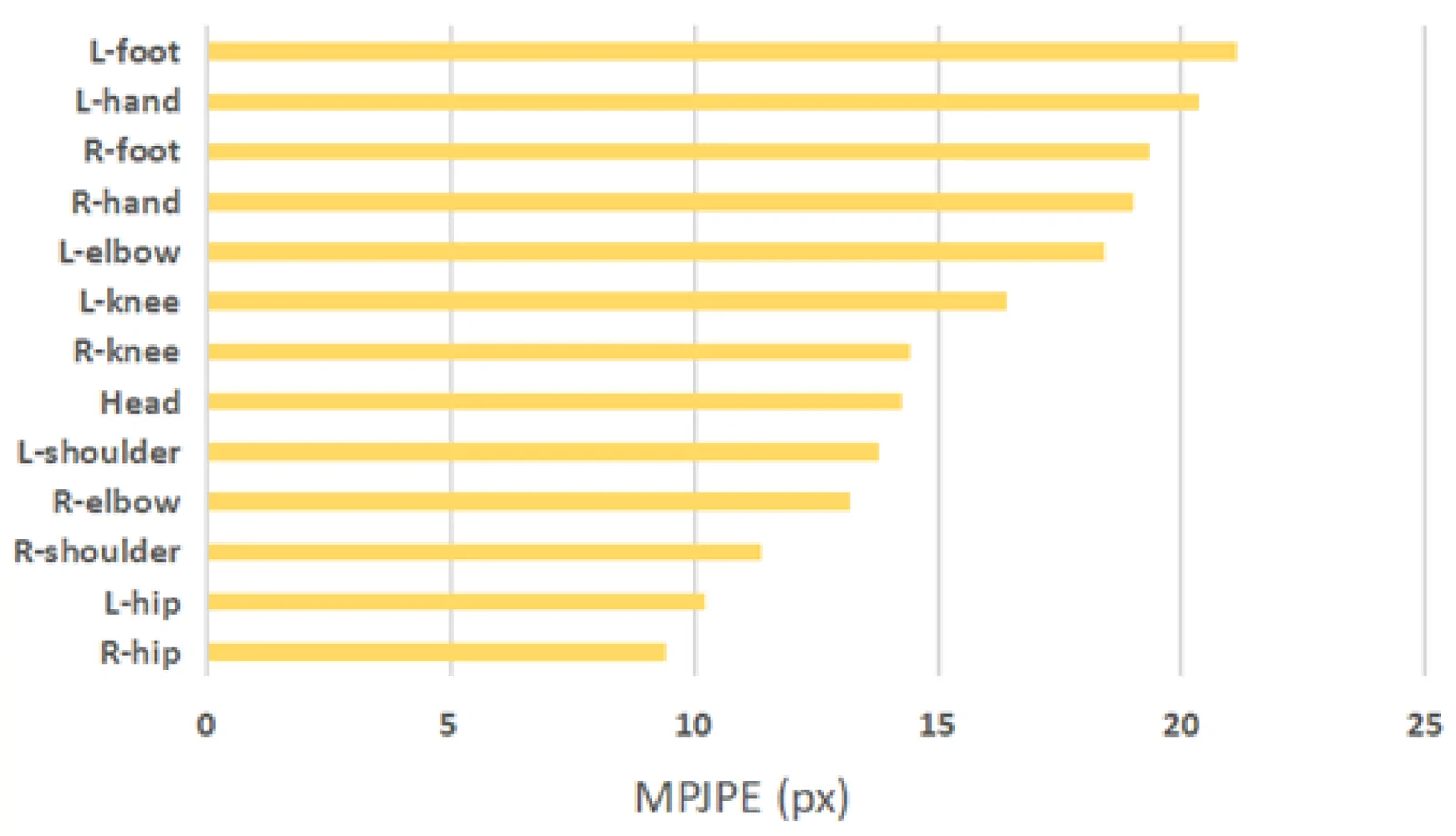
Per keypoint MPJPE sorted in descending order. Larger errors are mainly concentrated in hand and foot keypoints.

Both cases illustrate that the remaining errors are concentrated around flexible limb endpoints rather than the main torso structure. The current method processes each video frame independently and cannot use temporal continuity to stabilise short-term ambiguities. This observation is also consistent with the per-keypoint error distribution in Figure 6, where the hand and foot keypoints exhibit larger localisation errors.

To further identify the error sources, Figure 6 shows the MPJPE of each keypoint. Larger errors mainly occur at the left foot, right foot, left hand, and right hand, whereas the head, shoulder, and hip keypoints show lower errors. This distribution is consistent with the qualitative observations in Figure 5. The model reliably recovers the main body structure in simple and moderately difficult scenes, while fine localisation of endpoint keypoints remains more difficult in complex scenes because these regions are smaller, more flexible, and more likely to be affected by occlusion, weak boundary evidence, or local distractors.

### 4.2. Comparison with Baseline Methods

The proposed method was compared with several representative baselines. The first baseline is YOLOv8-Pose [31], which is a human pose estimation model trained under the COCO human keypoint setting. The COCO-pretrained model predicts 17 human keypoints, and the corresponding outputs were mapped to the proposed 13-keypoint robot protocol. The second YOLOv8-Pose model was fine-tuned directly on the robot annotations and evaluated with both full-image and detector-ROI inputs. HRNet-W18 was trained under the same 13-keypoint protocol and evaluated using the detector ROI. Its result with the ground-truth ROI was also included as an oracle reference. The ResNet18-based direct coordinate regression model trained using the proposed humanoid robot keypoint annotations. This baseline uses the same robot-specific annotation protocol and ROI input setting as the proposed method but removes the heatmap representation, DSNT decoding, and attention refinement.

The quantitative results are summarised in Table 10. The directly transferred YOLOv8-Pose baseline obtains an MPJPE of 201.50 pixels, an NME of 0.81, a PCK@0.20 of 0.17, and an OKS of 0.28. Its performance is much lower than that of the robot-specific methods. This large gap indicates a substantial domain and label-definition mismatch between the COCO human-pose setting and the present humanoid-robot task. Differences in rigid body surfaces, joint morphology, limb proportions, and local texture further limit direct transfer under the current evaluation protocol. Fine-tuning on the robot annotations reduces MPJPE to 24.97 pixels for full-image input and increases OKS to 0.928, showing the importance of task-specific supervision and a matched keypoint definition. Using the common detector ROI further reduces MPJPE to 18.85 pixels and raises OKS to 0.957, consistent with the benefit of suppressing irrelevant background and normalising target scale. The ROI-based YOLOv8-Pose model achieves the highest PCK@0.20 of 0.976, whereas the proposed model achieves lower MPJPE and NME and a higher OKS.

HRNet-W18 obtains an MPJPE of 30.82 pixels, an NME of 0.125, a PCK@0.20 of 0.856, and an OKS of 0.883 with the detector ROI. Replacing the detector ROI with the ground-truth ROI reduces MPJPE to 21.49 pixels and increases OKS to 0.936. This difference shows that HRNet-W18 is affected by crop quality under the present protocol. Because its MPJPE with the oracle ROI remains higher than that of the proposed model with detector ROIs, crop quality alone does not explain the observed performance gap; feature representation and coordinate decoding are also relevant factors. Compared with the ResNet18 direct regression baseline, the proposed method reduces MPJPE from 27.98 pixels to 15.49 pixels and improves OKS from 0.80 to 0.96. This improvement shows that localising the robot body alone is not sufficient for accurate pose estimation. Direct coordinate regression can learn the approximate robot body layout from a normalised ROI, but it has limited ability to model the spatial distribution around each keypoint. In contrast, the proposed method first learns heatmap-based spatial responses and then converts them into continuous coordinates through DSNT. This allows the model to exploit both local neighbourhood evidence and continuous coordinate supervision. The attention refinement module further suppresses irrelevant responses and enhances keypoint-related features.

**Figure 7:**
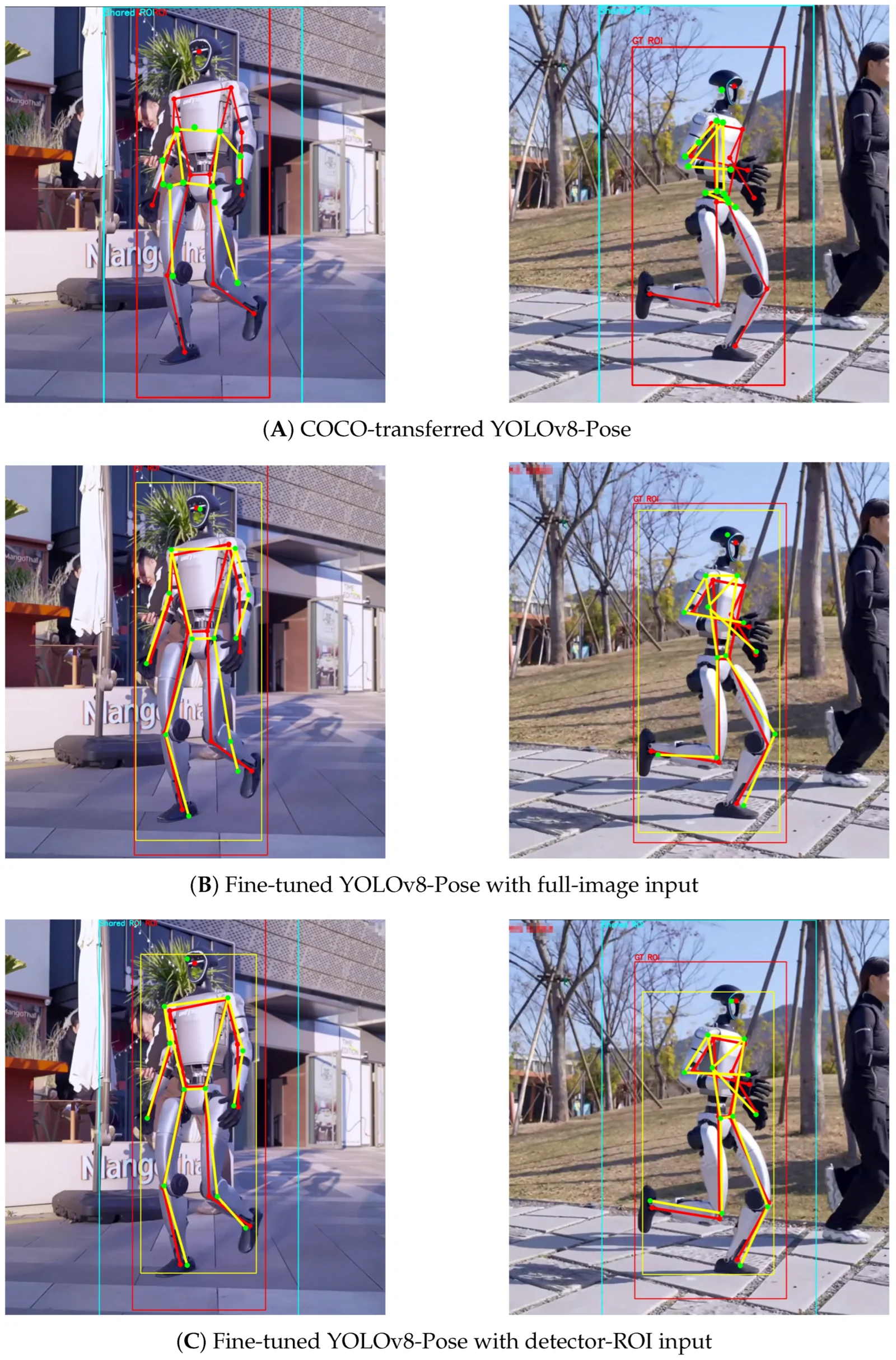

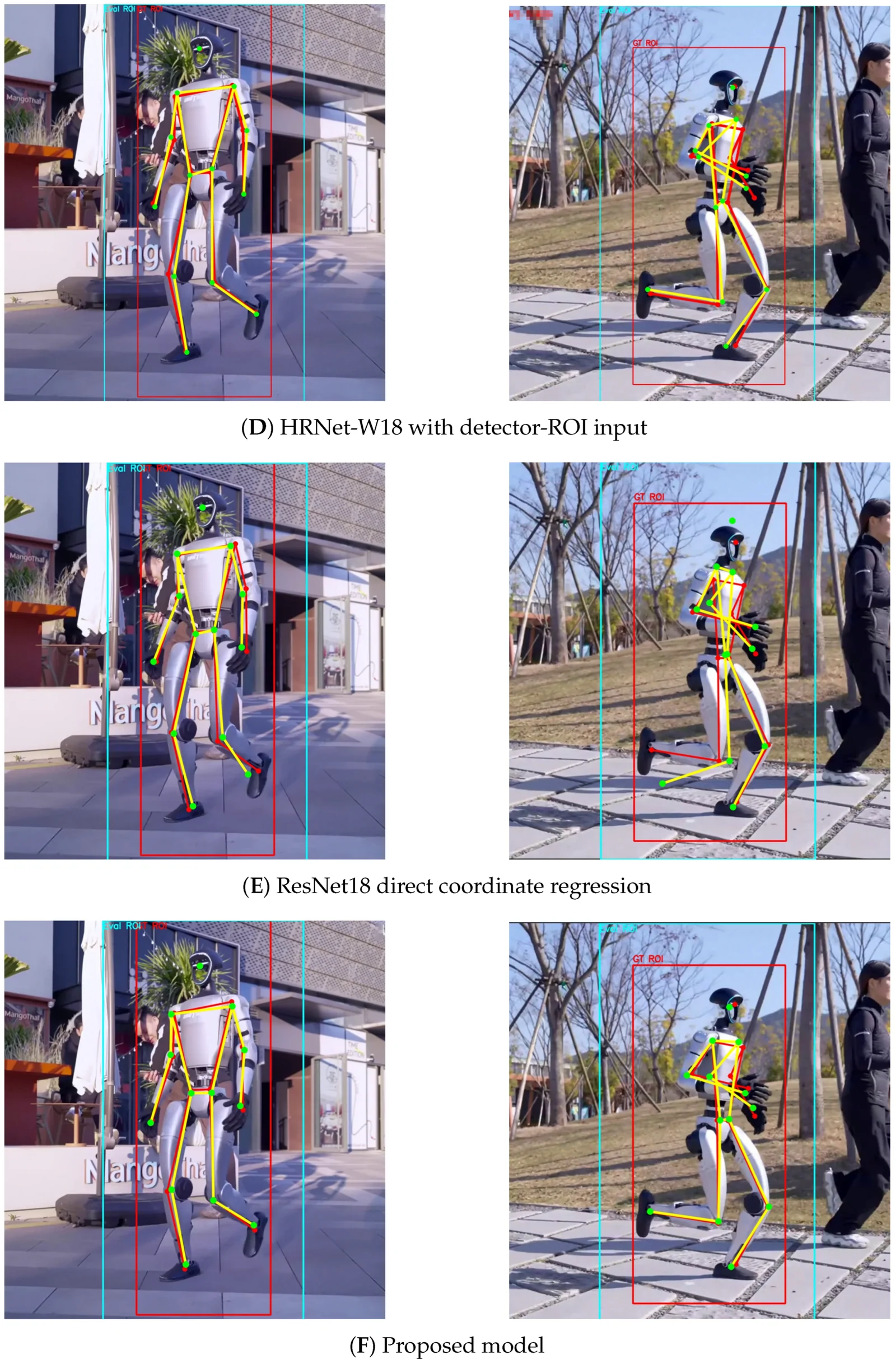
Visual comparison of representative baseline methods and the proposed model on the same test samples. (**A**) COCO-transferred YOLOv8-Pose; (**B**) fine-tuned YOLOv8-Pose with full-image input; (**C**) fine-tuned YOLOv8-Pose with detector-ROI input; (**D**) HRNet-W18 with detector-ROI input; (**E**) ResNet18 direct coordinate regression; (**F**) the proposed model. Red keypoints and skeletons denote ground truth, while green keypoints and yellow skeletons denote predictions. Visual comparison of representative baseline methods and the proposed model (continued). All methods are visualised on the same two test samples.

Representative visual comparisons are shown in Figure 7. The COCO-transferred YOLOv8-Pose model shows clear keypoint misalignment. Fine-tuning under the 13-keypoint robot protocol produces a more coherent skeleton, and detector-ROI input further reduces several endpoint offsets. HRNet-W18 recovers the main body structure, but larger deviations remain around the limbs. The direct regression baseline captures the approximate pose and still shows local structural distortion. The proposed method produces the most consistent alignment with the ground truth in both examples, especially around the hands and feet.

Reported results from other robot pose studies remain unsuitable for direct numerical comparison because their input settings, output spaces, datasets, and keypoint definitions differ from the present 13-keypoint protocol.

### 4.3. Component Ablation Analysis

A step-wise ablation study was conducted to examine the contribution of the main components. All models were trained and evaluated using the same data split, detector input, keypoint protocol, and evaluation metrics. The model configurations are listed in Table 11, and the quantitative results are shown in Table 12.

Model A is the ResNet18-based direct coordinate regression baseline. Model B retains the ResNet18 backbone and replaces direct coordinate regression with heatmap representation and Argmax decoding. DSNT is not used in Model B. Model C further replaces Argmax with DSNT and introduces coordinate supervision on the decoded continuous coordinates. CBAM is not included in Model C. Model D is the full proposed model, which adds CBAM-based feature refinement.

The largest gain in the sequential ablation occurs from Model A to Model B. Replacing direct coordinate regression with heatmap representation and Argmax decoding reduces MPJPE from 27.98 pixels to 18.75 pixels, a reduction of approximately 33.0%, while OKS increases from 0.80 to 0.93. Within this ablation sequence, the change in spatial keypoint representation therefore contributes the largest observed improvement. Heatmap supervision exposes local response structure that is absent from direct numerical coordinate output.

Replacing Argmax with DSNT further reduces MPJPE from 18.75 pixels to 16.61 pixels, corresponding to an improvement of approximately 11.4%. Argmax selects the maximum response on a discrete grid and is therefore limited by heatmap resolution, whereas DSNT computes a continuous coordinate expectation over the predicted spatial distribution.

The CBAM module provides a smaller additional gain. Compared with Model C, Model D reduces MPJPE from 16.61 pixels to 15.49 pixels and improves OKS from 0.94 to 0.96. Under the current dataset and training protocol, this result is consistent with attention-based refinement improving the discrimination of keypoint-relevant local features, although the gain is smaller than that associated with the change in spatial representation.

The additional variants isolate the effects of the auxiliary losses and training strategies. Removing the bone loss increases MPJPE from 15.49 pixels to 16.43 pixels, while PCK@0.20 decreases from 0.969 to 0.965 and OKS remains 0.964; thus, the structural term provides a modest improvement in localisation. Removing the shape loss increases MPJPE to 15.71 pixels and reduces OKS to 0.963. Larger degradations occur when training-time robustness strategies are removed: MPJPE rises to 25.97 pixels without ROI jitter and to 20.23 pixels without background replacement. Removing detector-guided cropping causes the largest degradation, with MPJPE increasing to 51.90 pixels, PCK@0.20 decreasing to 0.562, and OKS falling to 0.754. These results support the importance of local scale normalisation and crop perturbation under the present limited-data setting.

**Figure 8:**
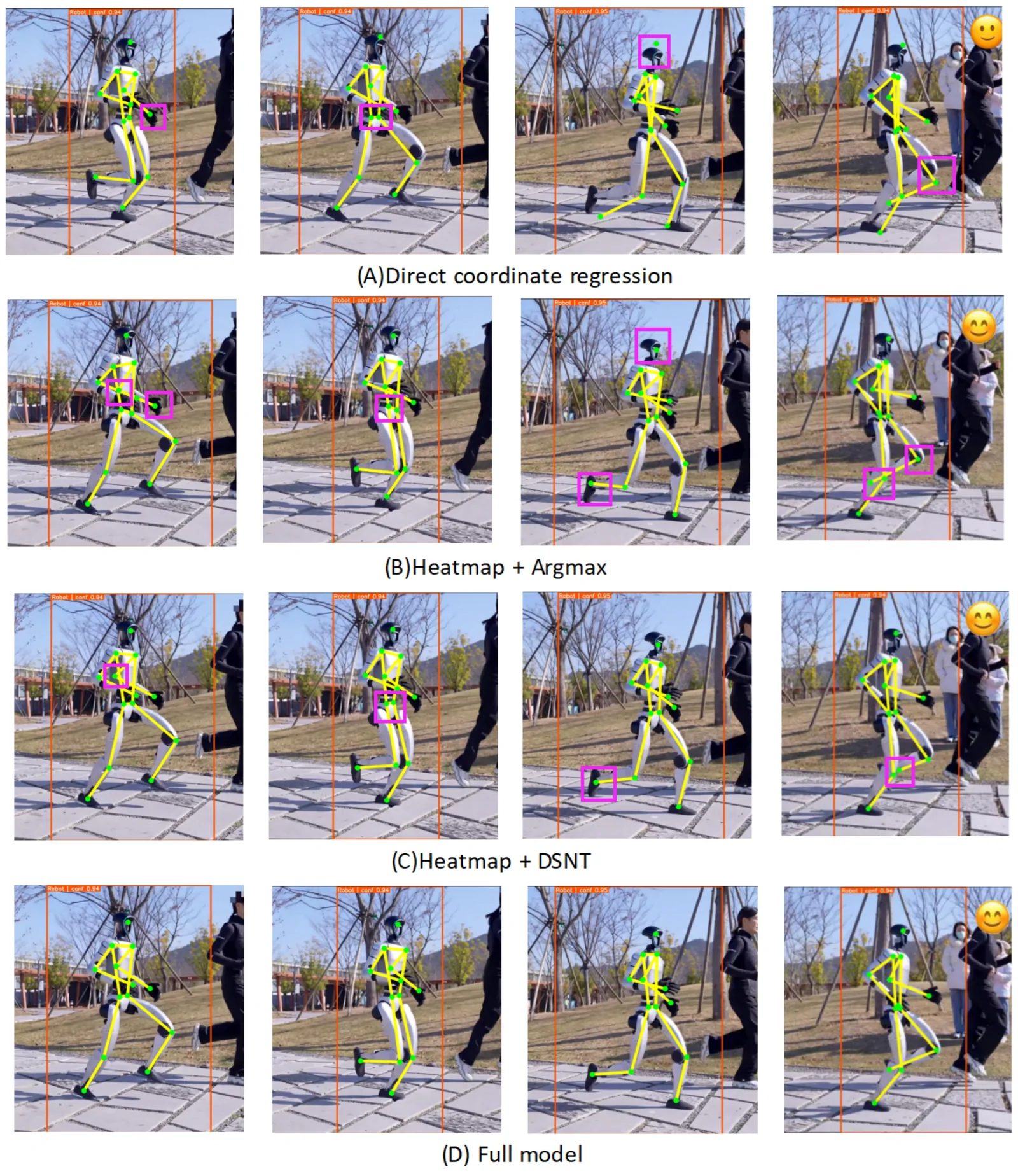
Qualitative comparison of ablation models on the same four frames. Red boxes mark keypoint regions or pose structures with noticeable differences.

The qualitative outputs of the ablation models are shown in Figure 8. The direct regression model is more likely to produce local keypoint shifts and skeleton distortion. Heatmap-based models produce more stable spatial responses, and the full model gives the most coherent skeleton structure among the compared settings.

### 4.4. Sensitivity to ROI Localisation Errors

Detector-guided cropping makes ROI quality an important factor in the complete pipeline. To isolate its influence, controlled perturbations were applied to the ground-truth ROI. The resulting crops cover several overlap levels. The achieved IoU between each perturbed crop and the corresponding ground-truth ROI was used for grouping. The trained Model D and all inference settings were kept fixed throughout this experiment. No retraining was performed. The ground-truth ROI serves as the ideal cropping reference.

Table 13 shows a clear performance decline as ROI overlap decreases. The ground-truth ROI produces an MPJPE of 13.43 pixels. For controlled crops with achieved IoU between 0.75 and 0.95, MPJPE is 14.48 pixels, while PCK@0.20 and OKS remain 0.969 and 0.968, respectively. Thus, the downstream pose estimator is relatively insensitive to small ROI perturbations under these controlled conditions.

The MPJPE of 14.48 pixels is slightly lower than the 15.49 pixels obtained by the original pipeline with detector-generated ROIs. These results correspond to different ROI sources. The high-IoU interval contains controlled crops generated from the ground-truth ROI. It does not represent the output of the detector. These crops retain high overlap with the annotated robot region and may be more regular than actual detector boxes. The two results therefore describe different evaluation conditions.

Performance decreases more clearly when the achieved IoU falls below 0.75. MPJPE rises to 31.78 pixels in the interval from 0.50 to 0.75, and PCK@0.20 decreases to 0.847. A stronger degradation occurs below an IoU of 0.50. MPJPE reaches 148.09 pixels for IoU values between 0.20 and 0.50. It further increases to 449.13 pixels when the IoU is below 0.20. Under this severe condition, PCK@0.20 falls to 0.032 and OKS decreases to 0.051. The large MPJPE standard deviations in the low-IoU intervals also indicate unstable keypoint predictions.

High-overlap crops generally preserve the complete robot body and maintain a suitable input scale. Lower-overlap crops may truncate limb regions, displace the robot within the normalised input, or introduce large irrelevant regions. The resulting appearance departs substantially from the crop distribution observed during training. Model D therefore tolerates minor ROI perturbations but degrades rapidly under severe localisation errors. Reliable front-end localisation remains necessary for stable operation of the complete detection-guided pipeline.

### 4.5. Sensitivity to Backbone Choice

The final local pose estimator uses ResNet18 as its feature backbone. To examine the sensitivity of the proposed architecture to this choice, Model D was retrained with MobileNetV2 and ResNet34. The detector-ROI input, 13-keypoint protocol, data split, and training schedule were kept unchanged. Each variant retained CBAM refinement, heatmap prediction, DSNT decoding, and the same loss design. Only the feature backbone and the channel dimensions connected to its output were adapted. Inference speed was measured on the same device using the complete detector-guided evaluation pipeline. Detailed comparison results are summarized in Table 14.

MobileNetV2 reduces the parameter count from 13.96 million to 8.33 million, but MPJPE increases from 15.49 pixels to 24.95 pixels and OKS decreases from 0.964 to 0.918. Thus, the more compact backbone does not preserve localisation accuracy in the present implementation. Its lower parameter count also does not translate into higher end-to-end FPS, which is plausible because the detector and ROI-processing stages contribute fixed cost and operator efficiency is hardware- and implementation-dependent.

ResNet34 achieves the best localisation accuracy, reducing MPJPE by 0.54 pixels relative to ResNet18 and increasing PCK@0.20 from 0.969 to 0.976. The deeper backbone provides greater representational capacity and a larger effective receptive field, which may help resolve weak limb textures and spatial relationships among visually similar joints. Because the gain is modest, these results are interpreted as a backbone-capacity trade-off rather than evidence that additional depth is uniformly beneficial.

The additional capacity of ResNet34 yields only a moderate numerical gain, while its parameter count increases to 24.07 million and end-to-end speed decreases to 2.36 FPS. ResNet18 uses approximately 42% fewer parameters and achieves the highest measured speed of 2.77 FPS, with accuracy close to that of ResNet34 across the reported metrics. ResNet18 was therefore retained as the final backbone for the accuracy–complexity trade-off observed in this implementation. MobileNetV2 remains the smallest variant by parameter count, although its localisation accuracy is lower.

## 5. Discussion

The experimental results show that the design of the keypoint recovery process has a direct influence on humanoid robot pose estimation. Both the baseline comparison and the ablation study demonstrate that moving from direct coordinate regression to heatmap representation substantially improves localisation accuracy and overall keypoint matching. Continuous coordinate decoding and attention-based feature refinement further provide consistent improvements. These results show that independent coordinate regression provides insufficient spatial representation for humanoid robot pose estimation. Stable localisation depends on discriminative local response maps and continuous coordinate recovery.

The extended ablation results further separate the effects of auxiliary losses and training strategies. Removing the skeleton-aware loss increases MPJPE from 15.49 pixels to 16.43 pixels, while removing the entropy-based shape loss increases it to 15.71 pixels. Larger degradations occur without ROI jitter (25.97 pixels), background replacement (20.23 pixels), or detector-guided cropping (51.90 pixels). Within the present ablation design, these results indicate that local input normalisation and training-time crop/scene diversification have a larger effect than the auxiliary regularisation terms.

The error distribution is not uniform across keypoints. Predictions for the head, shoulders, and hips are relatively stable, suggesting that the network can effectively model the main body structure of humanoid robots. Errors remain more evident for hands and feet, which are endpoint regions with higher motion freedom, smaller visual scale, and weaker appearance evidence. This indicates that the remaining difficulty lies mainly in fine endpoint localisation rather than global skeleton recovery. The pose-category analysis is consistent with this interpretation: walking poses achieve an MPJPE of 13.89 pixels, compared with 22.06 pixels for turning poses. The higher error for turning poses coincides with greater limb overlap and reduced endpoint visibility.

The baseline comparison also highlights the importance of robot-specific keypoint definition and training. Directly transferred YOLOv8-Pose performs poorly under the proposed 13-keypoint protocol. Fine-tuning on the robot annotations reduces MPJPE to 24.97 pixels for full-image input. Detector-ROI input lowers it further to 18.85 pixels. These results support the value of robot-specific supervision and scale-normalised local input. HRNet-W18 obtains an MPJPE of 30.82 pixels with the detector ROI and 21.49 pixels with the ground-truth ROI. Model D achieves the lowest MPJPE and NME and the highest OKS. The detector-ROI YOLOv8-Pose model obtains a slightly higher PCK@0.20. The remaining difference between HRNet-W18 with the ground-truth ROI and Model D shows that crop quality is only one factor. Feature extraction and continuous coordinate decoding also affect localisation accuracy.

From a deployment perspective, the proposed method uses only monocular RGB frames at inference. Compared with approaches that require multi-view reconstruction, depth sensors, motion-capture equipment, or explicit internal-state input, this setting reduces sensing and integration requirements. The present results show that useful full-body 2D keypoint estimates can be obtained under this constrained input setting, although broader cross-platform validation remains necessary.

The backbone comparison provides additional evidence for the choice of ResNet18 in the present implementation. ResNet34 achieves an MPJPE of 14.95 pixels, improving on ResNet18 by 0.54 pixels, but the experiment does not isolate which architectural property is responsible for this small gain. ResNet34 also increases the parameter count from 13.96 million to 24.07 million and reduces the complete-pipeline speed from 2.77 FPS to 2.36 FPS. MobileNetV2 contains only 8.33 million parameters, but its MPJPE rises to 24.95 pixels. Its measured speed is also lower than that of ResNet18. Operator implementation and the fixed cost of detection and ROI processing affect the end-to-end runtime. Under the evaluated implementation and hardware setting, ResNet18 therefore provides a more favourable balance between localisation accuracy, model size, and measured inference efficiency.

The method also has several limitations. First, the dataset remains relatively small, with 640 annotated images and a limited number of robot appearances and motion patterns. Although background replacement increases scene diversity during training, it cannot fully compensate for insufficient variation in robot body shape, pose configuration, viewpoint, lighting, and self-occlusion. Therefore, the reported performance should be interpreted as evidence of task-specific adaptation under the current dataset, rather than as a guarantee of broad cross-platform generalisation. Second, the present model operates on single frames and does not explicitly exploit temporal continuity. When a keypoint response is unstable in a single frame, information from neighbouring frames cannot compensate for the error. Third, the detection-guided framework remains sensitive to severe ROI localisation errors. Controlled perturbations show that the model remains stable when the achieved IoU is between 0.75 and 0.95. MPJPE increases to 31.78 pixels when IoU falls between 0.50 and 0.75. It reaches 449.13 pixels when IoU is below 0.20. These intervals were generated from perturbed ground-truth ROIs and do not describe the distribution of actual detector outputs. They isolate the sensitivity of the downstream pose estimator to crop quality. Minor ROI deviations are tolerated, while severe ROI localisation errors can invalidate the local pose estimate.

Future work should expand the dataset towards stronger occlusion, more extreme viewpoints, and additional robot appearances. Particular attention is required for hands and feet, where the current errors are concentrated. Temporal modelling can use neighbouring frames to stabilise uncertain endpoint responses. Detection reliability also requires further improvement. Low-confidence ROI recovery, adaptive crop expansion, and stronger subject discrimination within the local region may reduce failures caused by inaccurate boxes or nearby distractors. These extensions can improve practical robustness while preserving the monocular input setting.

## 6. Conclusions

This paper investigated a detection-guided local pose estimation pipeline for humanoid robot keypoint estimation from monocular video frames. A customised 13-keypoint protocol was designed to describe the full-body configuration of humanoid robots, and a robot-centred ROI-based estimation strategy was developed to reduce the influence of background clutter and target scale variation. The proposed method combines heatmap representation, differentiable coordinate decoding, attention-based feature refinement, and skeleton-aware optimisation to improve the accuracy and structural plausibility of 2D humanoid robot pose estimation.

Experimental results on the test set show that the final model achieves an MPJPE of 15.49 pixels, an NME of 0.06, a PCK@0.20 of 0.97, and an OKS of 0.96. Compared with the ResNet18 direct regression baseline, the proposed method reduces MPJPE from 27.98 pixels to 15.49 pixels. Under the unified 13-keypoint protocol, its MPJPE is also lower than that of fine-tuned YOLOv8-Pose with detector-ROI input and HRNet-W18, which obtain 18.85 pixels and 30.82 pixels, respectively. Direct transfer of the COCO-pretrained YOLOv8-Pose model produces a substantially higher MPJPE of 201.50 pixels. The sequential ablation study shows that introducing heatmap representation produces the largest observed reduction in MPJPE, while DSNT-based continuous coordinate decoding and attention refinement provide additional gains. The extended ablation results further show measurable sensitivity to the training losses and data strategies. Removing the skeleton-aware loss increases MPJPE to 16.43 pixels. Larger degradations occur without ROI jitter, background replacement, or detector-guided cropping.

Overall, this work provides a task-adapted pipeline for monocular humanoid robot keypoint estimation under limited annotated data. The method avoids additional depth sensors, motion-capture equipment, and internal joint-state input, which makes it compatible with external RGB observation pipelines. Its current evidence, however, is limited to the evaluated dataset and should not be interpreted as broad cross-platform validation. The backbone comparison supports the use of ResNet18 in the final model. ResNet34 reduces MPJPE by only 0.54 pixels but increases the parameter count from 13.96 million to 24.07 million and reduces the complete pipeline speed from 2.77 FPS to 2.36 FPS. Controlled ROI degradation further shows that small localisation errors can be tolerated when IoU remains above 0.75. Severe crop errors cause rapid performance degradation, and MPJPE reaches 449.13 pixels when IoU falls below 0.20. At the same time, broader validation on more robot platforms, more diverse scenes, and more challenging occlusion conditions remains necessary. Future extensions involving larger datasets, temporal modelling, and stronger ROI-level subject discrimination may further improve the robustness and practical applicability of the proposed approach.

## Figures and Tables

**Table 1:** Comparison of representative vision-based robot pose estimation methods.

Method	Input	Main Output	Robot Type	Dataset/Setting	Model or State Prior
Amini et al. [14]	Mono RGB	Multi-robot 2D KP	Humanoid	HumanoidRobotPose; RoboCup scenes	None
Cho et al. [15]	Head-worn stereo	Full-body 2D/3D pose	Humanoid	Task-specific stereo dataset	No internal-state input
HoRoPose [16]	Mono RGB	3D pose and state	Articulated	Synthetic and real benchmarks	Robot geometry; states estimated
CtRNet [21]	Mono RGB	Camera-to-robot 6D pose	Manipulator	Synthetic training; two real datasets	Robot model and joint states
RoboKeyGen [22]	Mono RGB	3D KP, pose, and joint angles	Manipulator	Simulation; real-camera evaluation	Robot model; states estimated
Heo et al. [23]	Mono RGB	Full-body 2D KP	Humanoid	11 robot types; sparse-data setting	None
Wang et al. [24]	Four-view RGB	Absolute 3D pose	Humanoid	Task-specific multi-view setting	CRF robot model
Young and Roberts [25]	Mono RGB	21 full-body 2D KP	Unitree G1	8410 images; 500 annotated with 21 KP	No model/state; platform-specific KP
Bultmann et al. [26]	Multi-view RGB	Allocentric robot pose	Mobile robot	Task-specific real-scenes	3D robot model
RoboPose [27]	Mono RGB	6D pose and joint angles	Articulated	Four-robot benchmarks	Known model; optional states
Ours	Mono RGB	13 full-body 2D KP	Humanoid	640 annotated images	None

Note: KP denotes keypoints. The last column indicates whether explicit robot geometry, a predefined robot model, or internal joint-state information is used. Platform-specific keypoint definitions are identified where relevant.

**Table 2:** Dataset split and number of annotated images.

	Train	Val	Test	Total
Number of images	400	100	140	640

Note: the training and validation subsets were split at a ratio of 8:2.

**Table 3:** Keypoint indices and names used in this study.

Index	Keypoint	Index	Keypoint
0	Head	7	Left hip
1	Left shoulder	8	Right hip
2	Right shoulder	9	Left knee
3	Left elbow	10	Right knee
4	Right elbow	11	Left foot
5	Left hand	12	Right foot
6	Right hand		

**Table 4:** Detector-level evaluation for ROI generation on effective labelled samples.

Effective Samples	No Detection	No Match	Matched	DetRate	Recall@IoU_0.20_
140	0	0	140	1.0000	1.0000

Note: The 140 effective labelled samples correspond to the entire test set. All test samples contain a humanoid robot with 13 annotatable keypoints and without full occlusion. “No match” indicates that the selected detection box has an IoU lower than 0.20 with the reference bounding box.

**Table 5:** Main ROI perturbation and training-stage augmentation settings.

Setting	Stage 0	Stage 1	Stage 2
Epoch range	1–10	11–50	51–80
ROI padding range	[0.15, 0.25]	[0.15, 0.45]	[0.17, 0.30]
Centre shift	0.05	0.10	0.04
Scale perturbation	0.15	0.35	0.18
Aspect ratio perturbation	0.06	0.12	0.06
Background replacement probability	0.20	0.25	0.00
Random occlusion probability	0.05	0.15	0.05
Occlusion area range	[0.02, 0.08]	[0.02, 0.12]	[0.02, 0.08]
Horizontal flip probability	0.50	0.50	0.50

**Table 6:** Loss weights used at different training stages.

Loss Term	Stage 0	Stage 1	Stage 2
$\lambda_{coord}$	1.00	1.00	1.00
$\lambda_{hm}$	0.14	0.10	0.06
$\lambda_{shape}$	$1\times 10^{-5}$	$5\times 10^{-5}$	$5\times 10^{-5}$
$\lambda_{bone}$	0.03	0.03	0.03

Note: Stage 0–2 correspond to the training-stage augmentation schedule in Table 5. The heatmap term denotes the weighted heatmap distribution loss, and the shape term denotes the entropy-based heatmap concentration constraint.

**Table 7:** Training and inference parameters of Model D.

Parameter	Setting
ROI expansion ratio	0.20 per side; 1.40× width and height
Heatmap size	64×64
DSNT coordinate convention	[0,1]×[0,1]; *x*: left to right; *y*: top to bottom
Optimiser	AdamW
Weight decay	$1\times 10^{-4}$
Learning rate	Stage 0: $2\times 10^{-4}$; Stage 1: $2\times 10^{-4}$; Stage 2: $1\times 10^{-4}$
Learning-rate scheduler	ReduceLROnPlateau; factor: 0.5; patience: 5; minimum LR: $1\times 10^{-6}$
Batch size	16
Number of parameters	13.96 M
Inference FPS	2.77

**Table 8:** Overall test-set performance of the direct coordinate regression baseline and the final model.

Method	MPJPE (px)	NME	PCK@0.20	OKS
Direct coordinate regression	27.98	0.11	0.85	0.80
Final model	**15.49**	**0.06**	**0.97**	**0.96**

Note: Best results are shown in bold.

**Table 9:** Performance across different robot pose categories.

Pose Category	Samples	MPJPE (px)	NME	PCK@0.20	OKS
Walking	112	13.89	0.058	0.978	0.970
Turning	28	22.06	0.081	0.931	0.941

**Table 10:** Quantitative comparison with representative baseline methods.

Method	Keypoint Protocol	Pose Input	MPJPE (px)	NME	PCK@0.20	OKS
YOLOv8-Pose (COCO transfer)	COCO 17-to-13	Full image	201.50	0.810	0.170	0.280
YOLOv8-Pose (fine-tuned)	Robot 13	Full image	24.97	0.100	0.915	0.928
YOLOv8-Pose (fine-tuned)	Robot 13	Detector ROI	18.85	0.076	**0.976**	0.957
HRNet-W18	Robot 13	Detector ROI	30.82	0.125	0.856	0.883
ResNet18 direct regression	Robot 13	Detector ROI	27.98	0.110	0.850	0.800
Ours	Robot 13	Detector ROI	**15.49**	**0.062**	0.969	**0.964**
HRNet-W18 (oracle)	Robot 13	Ground-truth ROI	21.49	0.086	0.930	0.936

Note: The HRNet-W18 oracle result uses the ground-truth ROI and represents ideal cropping conditions. It is excluded from the comparison between deployable settings. Bold values indicate the best deployable result for each metric.

**Table 11:** Configurations of the ablation models.

Model	Representation	Attention	Decoder	Coordinate Loss
A	Direct coordinates	–	–	✓
B	Heatmap	–	Argmax	–
C	Heatmap	–	DSNT	✓
D (Ours)	Heatmap	✓	DSNT	✓

Note: “–” indicates that the corresponding item is not included, while “✓” indicates included.

**Table 12:** Ablation results for model components and training strategies.

Model or Variant	MPJPE (px)	NME	PCK@0.20	OKS
Model A: direct coordinate regression	27.98	0.110	0.850	0.800
Model B: heatmap with Argmax, without DSNT	18.75	0.080	0.960	0.930
Model C: heatmap with DSNT, without CBAM	16.61	0.070	0.950	0.940
**Model D: full model**	**15.49**	**0.062**	**0.969**	**0.964**
D w/o bone loss	16.43	0.062	0.965	0.964
D w/o shape loss	15.71	0.063	0.965	0.963
D w/o ROI jitter	25.97	0.106	0.886	0.916
D w/o background replacement	20.23	0.081	0.922	0.940
D w/o detector-guided crop	51.90	0.211	0.562	0.754

Note: Models A–D and the first four Model D variants use the shared detector ROI. The last variant uses the full image resized to 256 × 256. Best results and model are shown in bold.

**Table 13:** Keypoint estimation performance under controlled ROI degradation.

ROI Condition	Achieved IoU Mean ± Std.	MPJPE (px) Mean ± Std.	NME	PCK@0.20	OKS
GT ROI reference	1.000±0.000	13.43±6.62	0.054	0.980	0.972
0.75≤IoU<0.95	0.846±0.056	14.48±6.76	0.058	0.969	0.968
0.50≤IoU<0.75	0.620±0.070	31.78±21.68	0.127	0.847	0.884
0.20≤IoU<0.50	0.334±0.087	148.09±98.27	0.598	0.404	0.481
IoU<0.20	0.094±0.054	449.13±129.50	1.817	0.032	0.051

Note: The degraded intervals were generated by perturbing the ground-truth ROI and were grouped according to the achieved IoU. They do not represent the distribution of detector outputs. The remaining metrics are reported as mean values over the valid crops in each interval.

**Table 14:** Performance, model size, and inference speed under different backbone choices.

Backbone	Parameters (M)	FPS	MPJPE (px)	NME	PCK@0.20	OKS
MobileNetV2	**8.33**	2.53	24.95	0.100	0.883	0.918
ResNet18 (Model D)	13.96	**2.77**	15.49	0.062	0.969	0.964
ResNet34	24.07	2.36	**14.95**	**0.060**	**0.976**	**0.967**

Note: All variants were trained and evaluated using the same detector-ROI protocol and 13-keypoint setting. The ResNet18 variant corresponds to the final Model D. FPS was measured using the complete detector-guided pipeline on the same device. Bold values indicate the best result in each column.

## Data Availability

The annotated data generated in this study are available from the first author and the corresponding authors upon reasonable request.

## Abbreviations

AvgPool	Average Pooling
CBAM	Convolutional Block Attention Module
COCO	Common Objects in Context
Conv	Convolution
DSNT	Differentiable Spatial to Numerical Transform
IoU	Intersection over Union
KL	Kullback Leibler
MaxPool	Maximum Pooling
MLP	Multi-Layer Perceptron
MPJPE	Mean Per Joint Position Error
NME	Normalised Mean Error
OKS	Object Keypoint Similarity
PCK	Percentage of Correct Keypoints
PCK@0.20	Percentage of Correct Keypoints at a Threshold of 0.20
ROI	Region of Interest
SL1	Smooth L1
YOLO	You Only Look Once
